# A Likelihood Approach to Estimate the Number of Co-Infections

**DOI:** 10.1371/journal.pone.0097899

**Published:** 2014-07-02

**Authors:** Kristan A. Schneider, Ananias A. Escalante

**Affiliations:** 1 Department MNI, University of Applied Sciences Mittweida, Mittweida, Germany; 2 School of Life Sciences, Arizona State University, Tempe, Arizona, United States of America; 3 Center for Evolutionary Medicine and Informatics, The Biodesign Institute at Arizona State University, Tempe, Arizona, United States of America; British Columbia Centre for Excellence in HIV/AIDS, Canada

## Abstract

The number of co-infections of a pathogen (multiplicity of infection or MOI) is a relevant parameter in epidemiology as it relates to transmission intensity. Notably, such quantities can be built into a metric in the context of disease control and prevention. Having applications to malaria in mind, we develop here a maximum-likelihood (ML) framework to estimate the quantities of interest at low computational and no additional costs to study designs or data collection. We show how the ML estimate for the quantities of interest and corresponding confidence-regions are obtained from multiple genetic loci. Assuming specifically that infections are rare and independent events, the number of infections per host follows a conditional Poisson distribution. Under this assumption, we show that a unique ML estimate for the parameter (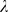) describing MOI exists which is found by a simple recursion. Moreover, we provide explicit formulas for asymptotic confidence intervals, and show that profile-likelihood-based confidence intervals exist, which are found by a simple two-dimensional recursion. Based on the confidence intervals we provide alternative statistical tests for the MOI parameter. Finally, we illustrate the methods on three malaria data sets. The statistical framework however is not limited to malaria.

## Introduction

Infections are ubiquitous and ecologically complex processes. Indeed the chain of events conducing to the colonization and replication of parasites within a host involves many environmental, physiological, and genetic factors both in the host and the infectious agent. A common observation in many host-parasite interactions is that there are multiple genetically distinct lineages of the pathogen infecting the same individual host [1]–[3]. Whereas in some diseases such as malaria, this is considered an important parameter, in others it is still somehow a neglected aspect that is just starting to be considered [2].

The observation of multiple genetic variants or multiplicity of infection (MOI) is indicative of the transmission dynamics since it allows for the co-transmission of different parasite variants or the overlap of several genetic variants due to multiple infectious contacts. Thus, the incidence of MOI or superparasitism per se is an important metric of exposure [2], [4]–[7]. In addition to its epidemiological importance, as many other ecological processes involving genetically distinct individuals, MOI leads to several outcomes derived from the interactions among lineages. This process is usually referred to as the intra-host dynamics [3].

During the last two decades, the outcomes of intra-host dynamics have been the subject of several theoretical and experimental investigations exploring a broad spectrum of scenarios. Usually, such studies focus on major effects that different interconnected factors have in terms of parasite dispersion (parasite fitness) and/or the elicited manifestations of disease that may lead to an effect on the host's fitness [3], [8]–[11]. Furthermore, intra-host dynamics also affect the spread of parasite lineages with adaptive mutations conferring resistance to antimicrobial agents or that allow the evasion of immune and/or vaccine-mediated protection [12], [13]. Under all these circumstances, following or measuring MOI as a parameter is essential whenever epidemiological inferences or models involving intra-host dynamics are formulated.

Although it is possible to control or measure the number of distinctive parasite lineages in models and experimental settings (e.g.[14]), a totally different scenario is the one faced by those studying naturally occurring infections in the context of ecological and epidemiological investigations [4]–[6], [15], [16]. Under such circumstances, MOI is usually measured by ad hoc metrics that rely on a set of genetic markers or the observed polymorphism in one or several genes [2]. The need for an experimental definition of MOI has generated approaches based on phylogenetic frameworks (e.g. many viruses) or some form of multi-locus genotyping [2], [17]. Whereas such approximations have been useful, there is still need for a formal statistical framework that allows the estimation of the actual number of lineages and other approximations to MOI that facilitates and/or considers confounding factors.

Given the broad spectrum of genetic architectures observed in parasitic organisms, it is not possible to define a universal framework of MOI. E.g. HIV accumulates mutations at a rate that allows for the use of phylogenetic base methods [17]. On the other hand, eukaryotic parasites such as *Plasmodium*, *Trypanosoma*, *Toxoplasma*, and *Schistosoma* [18], [19] and bacteria such as Mycobacterium [16] evolve at a rate at which it is possible to determine a stable number of genetically distinct lineages during the course of an infection given a set of genetic markers. In this investigation, we describe a formal statistical framework to estimate MOI that allows, among other aspects, building formal tests for comparing groups, e.g., before or after deploying an intervention such as a vaccine, complicated versus non-complicated cases, populations with different exposures, among other possibilities.

More specifically, we further develop the maximum-likelihood framework introduced by [20], which allows to estimate MOI and prevalences of pathogen lineages from a single genetic marker, e.g., microsatellite loci. We establish how to compute ML estimates and confidence intervals (or regions) for all involved parameters. Based on these, we show how statistical tests can be constructed to test the parameters. Although, the framework is - in principle - not restricted to a particular disease or species, we applied it to malaria by comparing data sets from three endemic regions with different levels of endemicity.

The philosophy behind the method section's structure is the following. We first establish the general methods and then refine them assuming that the number of co-infections follows a conditional Poisson distribution. This structure embraces a better understanding of how to derive particular results for alternative choices to the Poisson distribution. Moreover, rigorous mathematical proofs are shifted to the appendix. Readers less interested in these technical details should feel free to skip them.

## Methods

We adapt the maximum-likelihood method of [20] to estimate the average MOI. This approach is fully compatible with the model of [12], [21] which describes the hitchhiking effect associated with drug resistance in Malaria, for which MOI is a fundamental quantity. Being able to estimate MOI, the model can be ‘reverse engineered’ to reconstruct the evolutionary process underlying drug resistance. By doing so, a formal means is provided to identify those among the many compounding factors, which can be influenced to slow-down or prevent the spread of drug resistance in the course of public health initiatives.

### 1 Model background

Assume 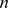 different ‘lineages’ 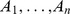 of a pathogen, e.g., 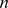 alleles at a marker locus (or haplotypes in a non-recombining region), circulate in a given population. Particularly, we have neutral markers in mind characterizing linages, so that their frequencies do not change too rapidly, e.g., due to selection. The 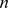 lineages considered are those that contribute to infection, not new variants that are generated by mutation inside hosts, but ‘fail’ to participate in transmission.

Because we identify a pathogen with the allele at the considered locus, we will use the terms ‘lineage’ and ‘allele’ synonymously. (We refrain from using the term strain, as we refer here to a genotypic characterization and the term strain may have different meanings across pathogens.)

In vector notation, the lineages' relative frequencies are 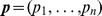. An individual (host) is infected by 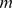 (not necessarily different) lineages of the pathogen with probability 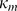. The 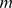 lineages are sampled randomly from the pathogen population. Hence, within an infection, the combination of pathogen linages follows a multinomial distribution with parameters 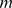 and 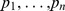. Consequently, the probability that 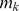 of the infecting linages carry allele 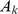 (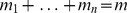) is given by 
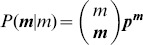
, where 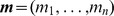, 
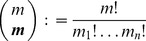
 is a multinomial coefficient, and 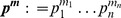. Clearly, 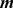 summarizes the pathogen configuration infecting a host.

In practice, 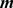 is unknown for a given host. It is possible to detect which alleles (or lineages) are present in a clinical sample, but it is difficult to reliably reconstruct 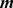 without using next generation sequencing, a technology that is not practical to use in many settings. For instance, if only a single allele, say 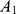, is found in a clinical sample, the patient might have been infected by just one parasite lineages (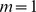), or co-infected by several lineages (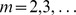), all of which carry allele 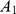. Hence, it is convenient to represent an infection (lineages detected in a patient) by a vector of zeros and ones of length 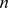, referring to the detected alleles (lineages). Hence, a clinical sample is represented by a vector 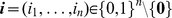, where 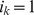 if 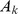 is found in the infection, and otherwise 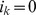. In mathematical terms 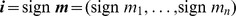. (Remember 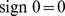 and 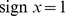 for 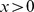). Note that the vector 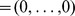 is excluded, which corresponds to no infection. In the following, 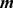 will always denote a vector of nonnegative integers and 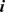 a vector of zeros and ones.

Let 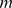 be the multiplicity of infection (MOI) with distribution 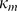. Because 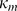 is unknown in practice, we aim to estimate it from clinical samples - or rather some summary statistics characterizing 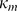.

Assume a total of 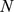 clinical samples, taken from different hosts roughly at the same time. We assume that the 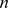 lineages 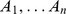 detected in the samples are all lineages circulating in the population. (There is no knowledge of undetectable lineages.) Each clinical sample contains one or more of the 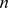 lineages (alleles). (We assume that lineages that infected the host have not vanished due to intra-host dynamics, e.g., drug treatments, and that new lineages have not emerged inside the host, e.g. by mutation, recombination etc.) A clinical specimen with allelic (or lineage) configuration 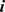 could descend from an infection with pathogen configuration 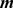 as long as 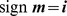. Let 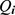 denote the expected frequency of clinical specimen with allelic configuration 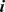. Then, 
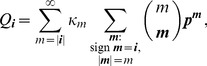
(1)where the first sum runs over all integers larger than or equal to 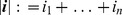, as this obviously is the minimum number of parasite lineages that could have caused the infection. The second sum runs over all possible configurations 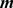 of exactly 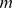 parasites that lead to the allelic configuration 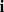 (i.e. 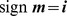), and hence could have potentially infected the host.

It follows, that for a given allele-frequency distribution 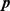, 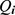 is determined by the distribution 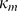. If infections with the pathogen are rare, a natural assumption is that the number of pathogens infecting a host is Poisson distributed, or more precisely follows a conditional Poisson distribution (CPD), i.e., 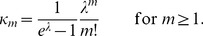
(2)


Of note, this conditions on the fact that each host is infected by at least one pathogen. The mean value of this distribution is 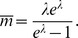



Assuming the CPD (2), 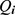 can explicitly be derived. In Analysis (subsection 4.1) it is shown that 
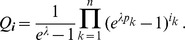



### 2 Maximum likelihood

Consider a total of 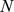 samples or clinical specimen, 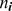 of which have allelic configuration 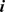. Hence, 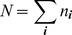, where the sum runs over all zero-one vectors of length 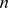, i.e,. 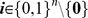 (the case of no infection i.e., 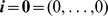 is excluded).

Since the (natural) likelihood for observing these samples is 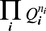, the log-likelihood is given by 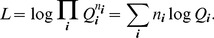
(3)


Assuming the CPD for the number of lineages infecting a host, it is shown in Analysis (subsection 4.2) that the log-likelihood becomes 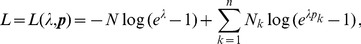
(4)where 
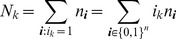
 is the number of samples that contain allele 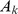. The prevalence of allele 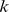 is then 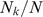. Notably, 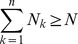 with equality if and only if exclusively single-lineage infections occur. This is one of two special cases that need to be treated separately. In the other special case all lineages are found in every infection. These cases are somewhat non-generic. We shall therefore formulate the following generic assumption.

**Assumption 1** *Assume that the sum over the alleles' prevalences is larger than one, but not all alleles are* 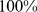 *prevalent. In other words, more than one lineage is found in at least one infection, i.e.,* 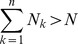 *and not all lineages are found in every infection, i.e.,* 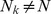 *for at least one* 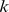 *.*

## Results

In the following 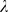 will refer to the parameter of the CPD, or in the general case, to the parameter (or parameter vector) summarizing the distribution 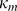. In the latter case 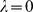 has to be interpreted as 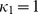.

We shall start by deriving the maximum likelihood (ML) estimates for the parameters of interest. Before we do so, we shall start by a rather intuitive observation.

Not surprisingly 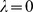 can never be an ML estimate if multiple alleles are found in at least one sample, as 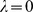 implies single infections only. We summarize this in the following remark which is proved in Analysis (subsection 4.3).

**Remark 1** *If at least one sample contains more than one allele, i.e.,* 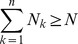, 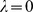 *is not the maximum likelihood estimate.*

To obtain the ML estimate for 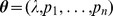, (4) needs to be maximized on the simplex, either using the method of Lagrange multiplies or by eliminating one of the redundant variables, i.e., by setting e.g., 
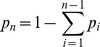
. When using Lagrange multipliers we need to find the zeros of the derivatives of 
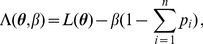
(5)i.e., 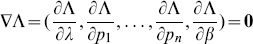. The derivatives based on the conditional Poisson distribution are derived in Analysis (subsection 4.4). The equations 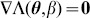 can be straightforwardly solved by a Newton method, i.e., by iterating



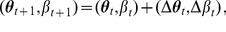
(6a)

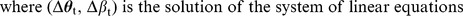


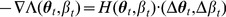
(6b)and 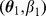 is any initial choice of 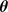 and 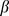. Here, 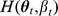 denotes the (transposed) Hessian matrix evaluated at 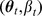, i.e.,



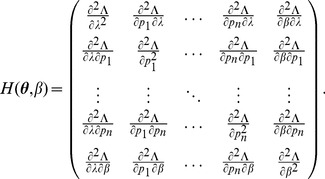
(7)If, in the general case, 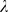 is a parameter vector, the derivatives above have to be interpreted accordingly.

In the case of the conditional Poisson distribution (2) the entries of the Hessian matrix are derived in Analysis (subsection 4.4).

Clearly, instead of (6) also 
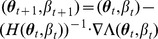
 can be iterated, which, however, is numerically less recommendable. Alternative approaches would be using an iterative least-square algorithm or the EM algorithm (cf. e.g.[22]).

Of note, in general, an ML estimate does neither necessarily exist, nor is it unique, not to mention that closed formulas typically do not exist. Unfortunately, assuming the CPD (2), the ML estimate indeed cannot be calculated explicitly. However, the estimate exists and is unique. Furthermore, although it can be straightforwardly derived by the above methods, the complexity of whole procedure can be greatly simplified.

**Result 1** *Assume the conditional Poisson distribution (2) for* 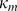 *. Under Assumption 1 there is a unique maximum likelihood estimate* 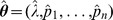 *. The first component* 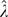 *is the unique positive solution of the equation.*

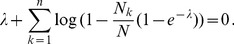
(8)


*It is found by iterating*

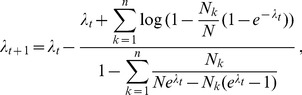
(9)
*which converges monotonically and at quadratic rate from any initial value* 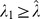.

*The maximum likelihood estimates of the allele frequencies are given by *

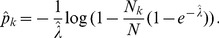
(10)


The result is proven in Analysis (subsection 5.1).

For the sake of completeness we shall also consider the instances in which Assumption 1 is violated. In the first situation, only one pathogen lineage is found in each infection, i.e., there is no indication whatsoever of co-infections. The results are summarized in the following remark which is proven in Analysis (subsection 5.1).

**Remark 2** *Assume that each sample contains only one allele, i.e.,* 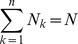 *. Then the ML estimates are* 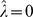 *and* 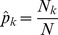 *.*

In the other non-generic case that all alleles are found in every sample an ML estimate does not exist, more precisely, it is 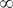, implying that – with probability one – all alleles are in every sample independently of the allele-frequency distribution.

**Remark 3** *Assume* 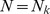 *for all* 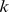 *. Then the ML estimate is “* 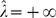 *” for every allelic distribution.*

A proof can be found in Analysis (subsection 5.1).

Of note, the maximum likelihood has an intuitive interpretation. We summarize this as the following result which is proven in Analysis (subsection 5.1).

**Remark 4** *The maximum likelihood estimate* 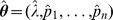 *is the set of parameters for which the observed number of samples containing allele* 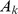 *equals its expectations, i.e., *

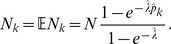



*Hence, the maximum likelihood maximizes the expectation of the log-likelihood.*

### 1 Confidence intervals from the profile-likelihood

Let 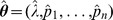 denote the ML estimate. Confidence intervals can be derived from the profile-likelihood for each parameter.

We are interested in finding a confidence interval (CI) for 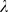. For a fixed value of 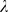, the profile likelihood is defined as 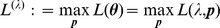
i.e., as the maximum likelihood taken over the remaining parameters while keeping the parameter of interest fixed. Moreover, denote the maximum likelihood by 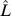 (clearly 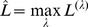). Suppose 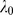 is the true parameter and 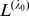 the corresponding profile likelihood. Then
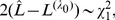
(11)i.e. twice the difference of the maximum likelihood minus the profile likelihood assuming the true parameter is 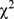 distributed with one degree of freedom (cf. e.g. [23], chapter 4). This can be used to construct confidence intervals for the true parameter 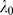. To construct a CI at the 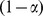 level, we need to find all 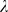 satisfying



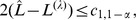
i.e., we need to find 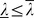 satisfying 
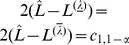
, where 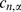 denotes 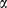-quantile of the 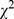 distribution with 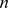 degrees of freedom. In other words, the equation 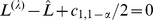 needs to be solved. By definition of 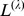, this means that 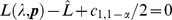 needs to be solved with respect to 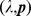, while simultaneously maximizing 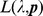 with respect to 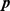. The latter is done using the method of Lagrange multipliers for fixed 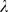, i.e.,



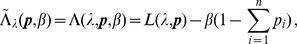
is maximized. This leads to the equations 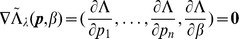. Therefore, following [24] the bound of the confidence intervals are found by solving the following system of equations
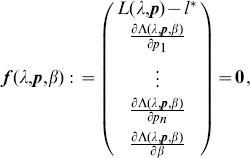
(12)where 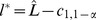


Clearly, 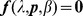 can be straightforwardly solved by a Newton method, i.e., by iterating 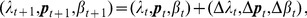
(13a)


where (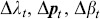) is the solution of the system of linear equations



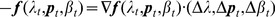
(13b)and 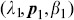 is any initial choice of 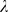, 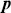 and 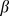. The derivative 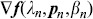 is identical to (7) except for the first line, which needs to be replaced by



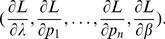
(14)The derivatives of 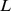 are given by (39). Hence, 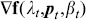 is given by 
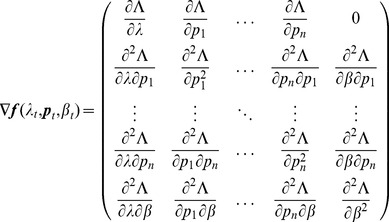
(15)where all derivatives are given by (39) and (40).

Again, alternatively 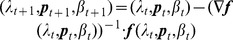 can be iterated, which however requires to invert the matrix 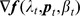 in every iteration step. The alternatives to the Newton method are again the EM algorithm or an iterated least-mean-square algorithm.

To obtain the confidence bounds 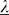 and 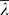 it is necessary to iterate (13) from two different initial values. Of note, obtaining one bound for the confidence interval is numerically only as demanding as obtain the ML estimate.

Confidence intervals for the allele frequencies 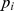 are obtained similarly by iterating (13) with obvious changes. Namely, the first component of the function 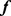 needs to be replaced by 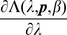 and the 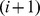-th component by 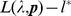, i.e., 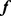 is the gradient of 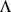 with the derivative with respect to 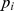 replaced by 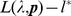. Consequently 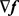 is identical to 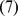 with the 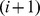-th component replaced by (14).

Importantly, existence and uniqueness of the confidence bounds 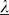 and 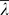 can be proved under the assumption of the CPD (2). Moreover, it is possible to significantly reduce the complexity of the Newton method (13) to find the CI's bounds. We obtain the following result, which is proven in Analysis (subsection 5.2).

**Result 2** *Suppose Assumption 1 holds. If* 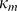 *is given by the conditional Poisson distribution (2), the confidence interval for* 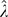 *(based on the profile likelihood) is uniquely defined.*

*The bounds of the confidence interval (* 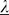 *and* 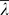 *) for* 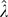 *are obtained by iterating*

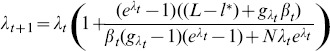
(16a)

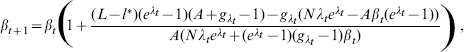
(16b)


*where*

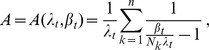


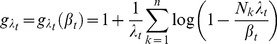
(16d)
*and*



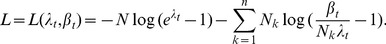
(16e)
*There are exactly two possible solutions* 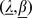 *and* 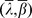 *. The algorithm is converging quadratically for any initial values* 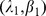 *sufficiently close to the one of the solutions.*

The proof is found in Analysis (subsection 5.2).

Formally, the above result holds true in the non-generic cases 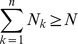 and 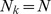. If all samples contain just one lineage, i.e., 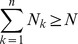, the ML estimate is 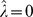 and the confidence interval has the form 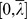. If all samples contain all lineages, i.e., 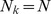 the maximum likelihood estimate is 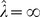 and the confidence interval has the form 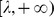, hence it is infinitely large. Although, formally the result still holds, the asymptotic (11) is no longer true, as discussed in Analysis (subsection 6), rendering the result inapplicable if Assumption 1 is violated.

### 2 Asymptotic confidence intervals

As an alternative to the profile likelihood, one can use the asymptotic normality of the maximum likelihood to construct confidence intervals. Asymptotically the difference of the maximum likelihood (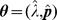) and the true parameter (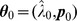) is normally distributed. However, it is important to notice that - unless one eliminates one of the redundant allele frequencies - the Lagrange multiplier 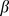 needs to be treated like a regular parameter. The corresponding likelihood function is of course given by (5). Hence, the actual parameters involved are 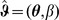. The difference of the maximum likelihood 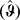 and the true parameter 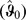 is asymptotically distributed according to 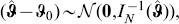
(17a)


or
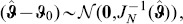
(17b)where 
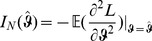
 is the expected Fisher information and 
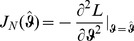
 is the observed Fisher information (based on sample size 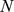). The matrix 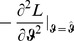 is the transposed Hessian matrix given by (7).

The expression 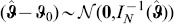 is the convenient, although imprecise notation, for 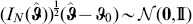, where 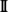 is the 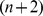-dimensional identity matrix and 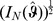 the symmetric square root of the Fisher information. Namely, any positive semi-definite, symmetric matrix 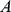 (as it is the case of any covariance matrix, and particularly the Fisher information) has a spectral decomposition 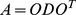, where 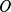 is orthogonal and 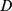 is the diagonal matrix that contains all eigenvalues. These are real and nonnegative, and the diagonal matrix that contains the square roots of the eigenvalues is denoted by 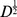. Hence, by setting 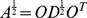, we have 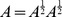.

An often used alternative notation is 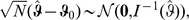
or



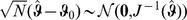
with 
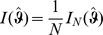
 and 
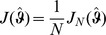
.

From (17) the asymptotic distribution of the parameters of interest 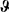 follows immediately by dropping the ‘dummy’ variable 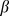 and the corresponding rows and column in the inverse Fisher information. Of note, this is not identical to ‘formally’ derive the inverse Fisher information based on 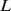 and 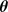. Namely, it is important to drive the asymptotic covariance matrix with respect to 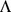 and 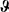.

Since 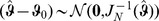 the bounds for the 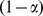 CI for 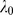 are given by 
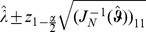
(18)and those for the components of 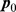 by
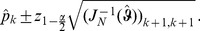
(19)


Here, 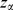 denotes the 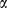 quantile of the standard normal distribution.

Of course, when using the expected Fisher information, 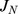 needs to be replaced by 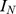. Under the assumption of the conditional Poisson distribution (2), the second derivatives 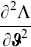 needed to derive the Fisher information are calculated in Analysis (subsection 4.4; eq.39). Moreover, evaluated at the maximum likelihood estimate, 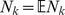, it is seen that the expected and observed Fisher information are identical, i.e., 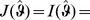, when assuming (2).

With some algebraic manipulation it is possible to simplify the expressions for the confidence intervals assuming the CPD (2).

**Result 3** *Suppose the number of co-infections follow the conditional Poisson distribution* (2) *and that Assumption 1 holds. Then an asymptotic* 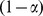 *-confidence interval for* 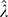 *is given by *

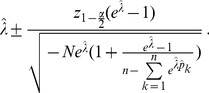
(20)


*Alternatively, the following formula, requires just the ML estimate for* 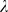


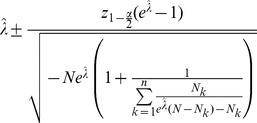
(21)


For a proof, see Analysis (subsection 5.3).

In the non-generic case 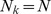 for all 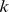, the ML estimate is not unique, and we have 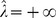. Hence, asymptotic CIs make no sense in this case, neither for 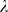 nor for the frequencies 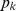.

In the case 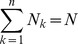, it also impossible to derive CIs as the asymptotics (17) break down (cf. subsection 6 in Analysis).

Explicit formulas for the CIs of the allele frequencies are obtained similarly.

**Result 4** *Under the same assumptions as Result 3, an asymptotic* 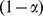 *-confidence interval for* 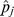 *is given by *

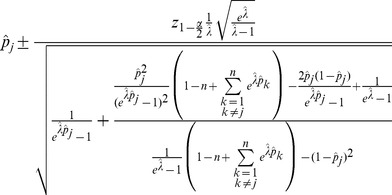
(22)


The proof can again be found in Analysis (subsection 5.3).

### 3 Testing the parameters

In practice, data from several loci is typically available, each of which yields a different ML estimate or there might be some prior estimate for the parameters of interest. Depending on particular properties of the marker loci (mutation rate, allele-frequency spectrum, biochemical issues in determining motif repeats, etc.) different marker loci will lead to different ML estimates. Hence, it is desirable to test whether different estimates are significantly different. The confidence intervals can be adapted to test the parameters.

Clearly, at different marker loci, different alleles will segregate and the allele-frequency spectra will be very different. Hence, for the present purpose, it is meaningless to compare the allele frequencies at different loci. However, the estimate for 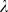 should be consistent, as this parameter is the same for all loci. Consequently, in the following we will focus on testing 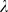 and present three alternative tests for the null hypothesis 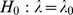 *vs.* the alternative 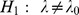.

#### 3.1 The likelihood-ratio test

The first test is rather straightforward. Since 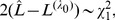
(23)under the null hypothesis 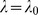, it is rejected at significance level 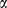 if



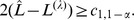



In other words, we reject the null hypothesis for any 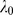 that lies outside the 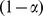-confidence interval of 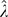, which are obtained as outlined above in “Confidence intervals from the profile likelihood”. Therefore, this test requires no additional numerical effort if the confidence intervals were already derived.

The corresponding p-value is given by 
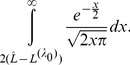
(24)


To calculate the p-value, 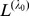 needs to be derived first. Similarly as in section in “Confidence intervals from the profile likelihood”, this leads to the equations 
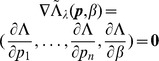
. Therefore, the system of equations 
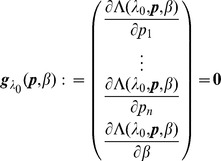
(25)needs to be solved by a Newton method, i.e., by iterating



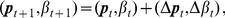
(26a)

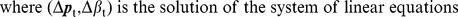


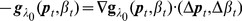
(26b)and 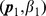 is any initial choice of 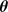 and 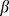. The derivative 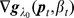 is obtained from (7) by deleting the first row and column and substituting 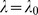, i.e.,
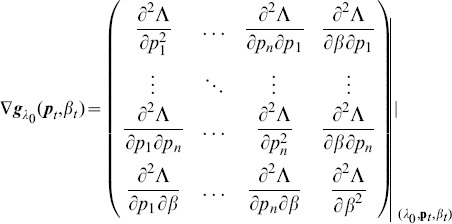
(27)where all derivatives are given by (39) and (40).

**Result 5** *Suppose Assumption 1 and* 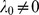 *holds. In the case of the conditional poisson distribution, the p-value under the null hypothesis* 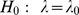 *is given by* (24), *where* 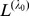 *is given by* (4) *with* 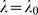 *and* 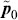 *given by *

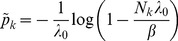
(28)
*where* 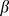 *is the solution of* (16e) *with* 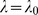.

*The solution* 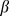 *is found by iterating *

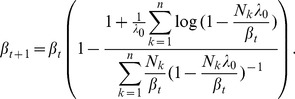
(29)


The proof is presented in Analysis (subsection 5.4).

In case of 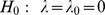, there are two possibilities. If 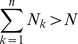, then 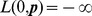. Hence, the null hypothesis is always rejected. This is clear, because if 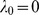 is the true parameter, it is impossible to observe data 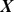 with 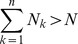 (see Remark 7 in Analysis, subsection 6). However, if 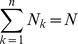, then 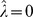 and 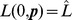, and the null hypothesis is always accepted.

Therefore, in the case of 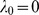 the test can still be formally performed in a meaningful way. However, note that the asymptotic (23) does not long hold true, as 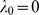 does not lie in the interior of the parameter space.

#### 3.2 The score test

In the following, for any parameter choice 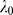, let 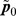 by the corresponding profile-likelihood estimate, i.e., 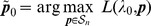, where 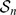 is the 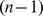 dimensional simplex. By using a dummy variable as before, 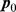 is obtained from 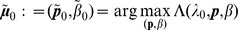. The Fisher information can be written as 
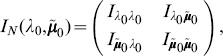
where 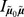 is obtained from the Fisher information with the first row and column deleted. The definitions of the remaining sub-matrices follow accordingly.

A test for the null hypothesis 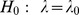 *vs.* the alternative 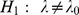 is obtained by using the fact that 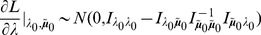
(30)(cf. Remark 6 in subsection 5.4 of Analysis). The function
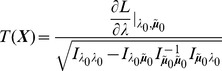
(31)serves as test statistic, where the data is 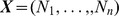. The test rejects 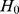 at the 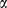-level if 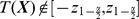 The corresponding p-value is 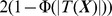.

Note that it is legitimate to write 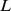 on the left-hand side of (30) because 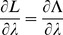. However, it is nevertheless important to derive the asymptotic variance from 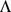.

Alternatively, the expected Fisher information 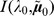 in (30) and (31) can be replaced by the observed Fisher information 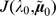. However, if 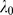 is not the ML estimate, 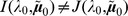. As proven Analysis (subsection 5.4), one obtains for the CPD:

**Result 6** *Consider the score test for the null hypothesis* 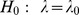 *vs. the alternative* 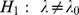 *under the assumptions of Result 5. The test statistic based on the observed Fisher information is *

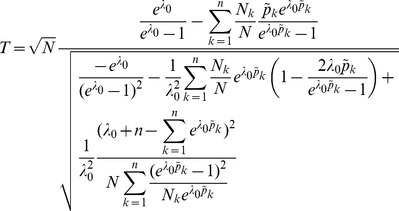
(32)
*and that based on the expected Fisher information is*



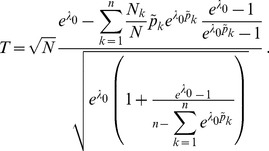
(33)
*The p-values are* 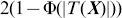 *in either case. The frequencies* 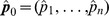 *are derived as specified in Result 5.*

Of note, instead of (30) the ML estimate can be used as a plug-in estimate for the asymptotic variance, i.e., 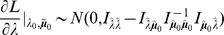. In this case, it is not necessary to distinguish between the expected and observed Fisher information as they coincide (cf. section “Asymptotic confidence intervals”).

In summary one obtains:

**Remark 5** *Under the assumptions of Result 6, a test statistic for the null hypothesis* 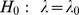 *vs. the alternative* 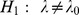 *is *

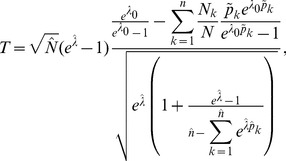
(34)
*where* 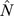 *and* 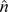 *are sample size and number of alleles, in the data yielding the estimate* 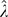.

The proof is analogously to the one of Result 6.

The test cannot be applied in the special cases 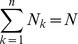 or 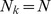 for all 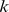, as the asymptotic (30) no longer holds true (cf. subsection 6 of Analysis).

#### 3.3 The Wald test

A third test for the null hypothesis 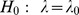 is an adaptation of the Wald test for the profile likelihood. It is based on the same asymptotic properties that we used to derive confidence intervals namely 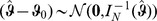. This is exactly the same as the asymptotic 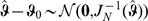 as 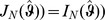.

This implies 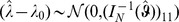 or 
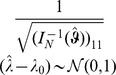
. Hence, the test statistic 
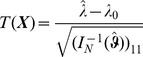
can be used. The p-value is 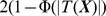.

Now, we shall consider again the CPD. An explicit expression for 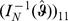 is given by (54). Hence, we obtain:

**Result 7** *Under the assumptions of Result 5, the Wald test for the null hypothesis* 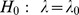 *vs. the alternative* 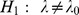 *has the test statistic *

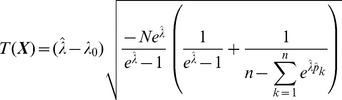
(35)
*based on the (expected or observed) Fisher information.*

*The p-values are* 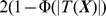 *in either case. Here,* 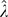 *and the frequencies* 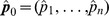 *are derived as specified in Result 1.*

Alternatively, if the profile-likelihood estimate based on 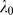 is used as a plug-in for the asymptotic variance, one can employ 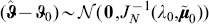 or 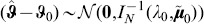.

In the first case, using (53) implies that the test statistic changes to 
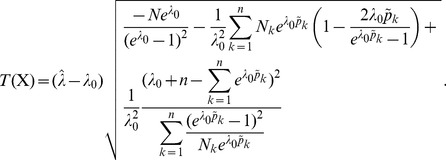
(36)


In the second case, (54) implies that the test statistic changes to 
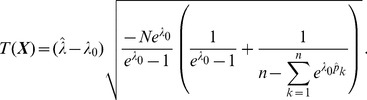
(37)


Also the Wald test cannot be applied in the special cases 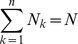 or 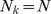 for all 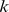, as the asymptotic for 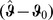 no longer holds true (cf. subsection 6 of Analysis).

### 4 Testing the method

Although - as we have seen - most of the theory works quite general, assuming a CPD for the number of co-infections permits to derive explicit results or, at least, reduces the complexity significantly. However, assuming a CPD might not be justified. Therefore, it is desirable to have a test for the model's fit. Namely, let 
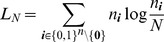
be the likelihood assuming a perfect fit to the data, in which the expected frequencies of infection with stain configuration 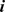 equal their observed frequencies. In other words, 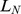 is the maximum likelihood of the saturated model. As there are 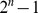 possible allelic configurations 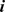 infecting a host, 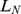 has 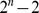 degrees of freedom. The maximum likelihood 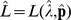 of the reduced model (assuming the CPD) has 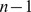 independent allele frequencies and one Poisson parameter. Therefore,



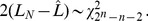
(38)Hence, the following test can be used.

**Result 8** *To test* 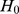 *: “the conditional Poisson distribution is justified” vs.* 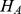 *: “the conditional Poisson distribution is not justified”, the test-statistic* 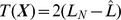 *can be used. The p-value is given by .* 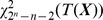


It should be mentioned that the above test might perform poorly if the number of lineages or alleles 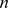 is large. The reason is that the 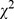 distribution has too many degrees of freedom. This might be the case when using hyper-mutable microsatellite markers with 10 or more alleles found across samples.

## Application to data

As an illustration, the methods are applied to three previously-described data sets [25]–[27]. Each of which comprises molecular data from *P. falciparum*-infected blood samples from endemic areas with different levels of malaria incidence. For each blood sample, parasite DNA was extracted and several microsatellite markers assayed.

### 1 Preliminary remarks

It is important to note beforehand that only (selectively) neutral markers should be included in the analysis. Namely, loci linked to others that are targets of selection (e.g., *mdr1*, *crt*, *dhfr*, *dhps* in *P. falciparum* that are associated with selection for drug resistance) will have skewed allele-frequency distribution. Hence, using these markers might lead to artifacts and severe misinferences. In practice, a marker located on a chromosome not carrying a strongly selected gene (e.g. resistance-conferring gene), can be regarded to be neutral. Moreover, clinical samples from groups that will be compared need to consider confounding effects such as differences in treatment polices, control interventions, and changing transmission intensities (e.g., a group should not contain samples from two time points during which treatment policies changed). By not considering such effects, the estimates of MOI would be inappropriate. For these reasons, we only used parts of the available data sets.

### 2 Data description

The first data set emerged from a longitudinal study conducted in Asembo Bay, a hyper-endemic region in Kenya, and was described in [27]. We included five (neutral) microsatellites on chromosome 2 and four (neutral) markers on chromosome 3. Additionally, we included two markers on chromosome 8, quite close to *dhfr*, which are common to all three data sets and meet Assumption 1. Only blood samples collected in the first study year (mid 1993 to mid 1994) were included, resulting in 42 blood samples.

The second data set described in [26] is from a study from Yaoundé, Cameroon, a region of intermediate/high transmission. Besides the two markers on chromosome 8 mentioned above, we included all eight available (neutral) microsatellite markers on chromosomes 2 and 3 from all 331 blood samples (data of one of the 332 original samples was unavailable).

The third data set is from Bolivar State, Venezuela, a region of low transmission. It was described in [25] and consists of 97 blood samples. Due to the low transmission intensities, for most markers each blood samples contains only one allele, violating Assumption 1. We included all markers that met Assumption 1 as well as all available neutral markers. Particularly, we included four on chromosome 2 and three on chromosome 3, two markers on chromosome 8 and one on chromosome 4, which are sufficiently distant from respectively *dhps* and *dhfr* to be considered neutral, and the two makers on chromosome 4, which were also included in the other data sets. All 97 blood samples were used.

### 3 Results

**Figure 1:**
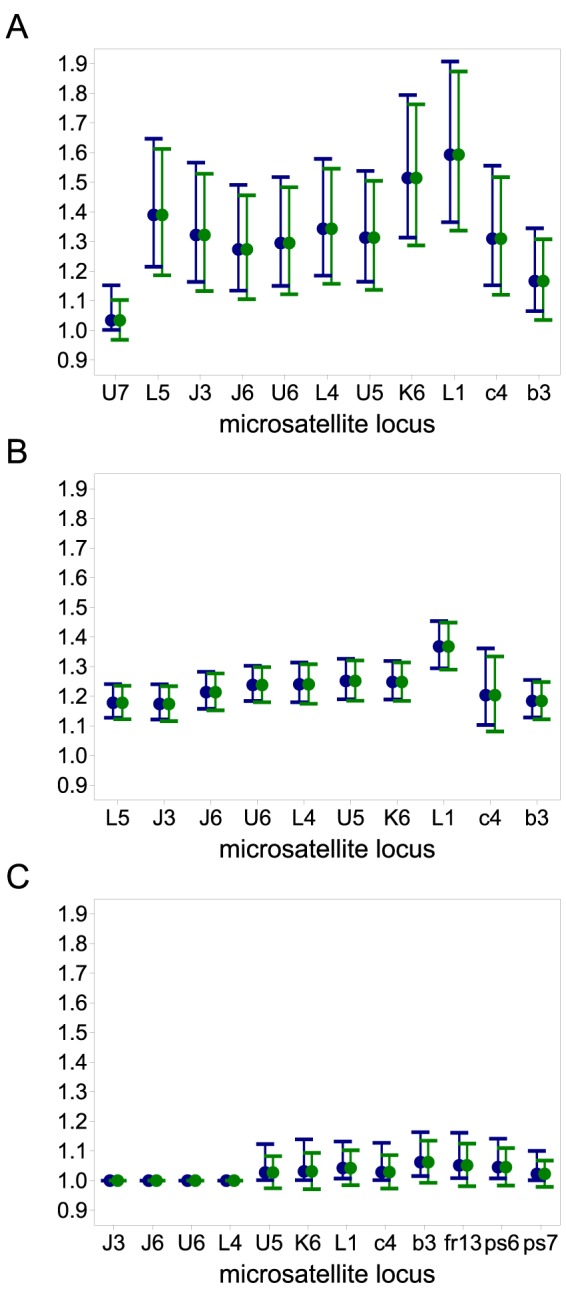
Shown are the ML estimates 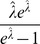 (dots) and their respective profile-likelihood-based (blue) and asymptotic (green) CIs for the data from Kenya (A), Cameroon (B) and Venezuela (C) for several microsatellite markers each.

**Figure 2:**
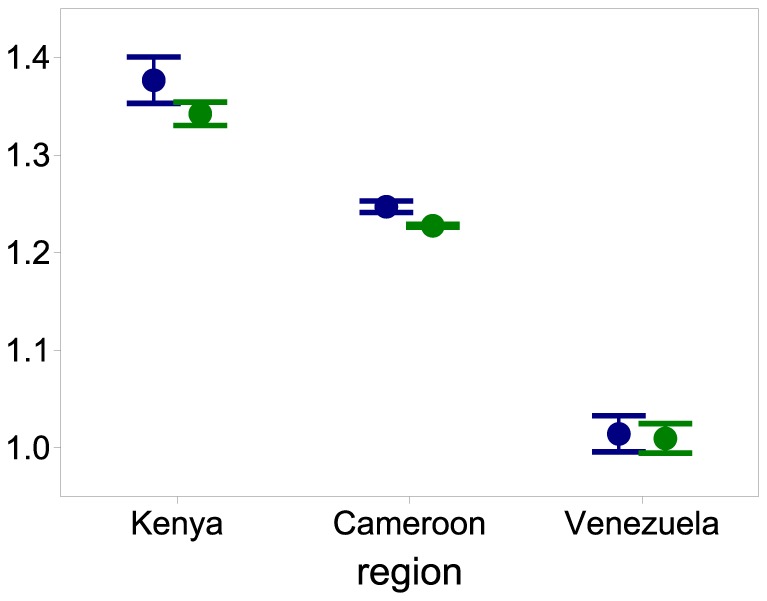
Average ML estimates by region. Averages are the arithmetic mean of the ML estimates 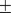 2 standard deviations derived from the microsatellite loci, which are common to all data sets, including (blue) and excluding (green) locus L1, which appears to be hyper-mutable in Kenya and Cameroon.

The results are summarized in Figures 1 and 2 and Tables 1–3. In all cases, the test for the model fit (cf. Result 8) justified the assumption of the CPD (cf. Tables 1–3). This is important because the three locations exhibit different transmission intensities. In all three regions, the ML estimates 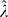 or rather the mean MOI, 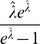, obtained from different marker loci are fairly consistent. As expected, most variation in the estimates is observed in Kenya because of the low sample size. Moreover, the transmission intensities are stronger, which leads to more variation in allele-frequency spectra among marker loci, resulting in more variation among the ML estimates.

**Table 1:** Estimates for each locus of the data set from Kenya.

locus	lower bound	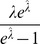	upper bound	2(*L_N_*–*L*_1_)	d.f.
U7	1.00194	1.03409	1.15244	6.40471	9
	0.968395		1.10265		
L5	1.21506	1.38975	1.64696	67.8528	15
	1.18622		1.61235		
J3	1.16387	1.32208	1.56625	44.993	16
	1.1331		1.52876		
J6	1.13457	1.27344	1.49108	58.3296	15
	1.10558		1.45595		
U6	1.15044	1.29506	1.51735	65.1444	14
	1.12211		1.48319		
L4	1.18509	1.34319	1.57899	89.2578	18
	1.15735		1.54568		
U5	1.16453	1.31318	1.53811	76.1215	20
	1.13692		1.50489		
K6	1.31334	1.51443	1.7943	134.024	26
	1.28687		1.76291		
L1	1.3654	1.59303	1.90742	87.4142	16
	1.33699		1.87367		
c4	1.15248	1.30977	1.55585	15.9715	7
	1.12049		1.51705		
b3	1.06529	1.16656	1.34475	34.7327	16
	1.03537		1.30777		

Each row shows, locus name, lower profile-likelihood (top) and asymptotic (bottom) confidence bound, ML estimate, upper profile-likelihood (top) and asymptotic (bottom) confidence bound. For the confidence, bounds *α* = 0.05 was assumed. Moreover, the test statistic for the fit of the CPD (2) is shown as well as the corresponding degrees of freedom. In all cases, the outcomes are not significant, suggesting that the assumption of the CPD is justified.

**Table 2:** See description of Table 1 but for the Cameroon data set.

locus	lower bound	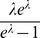	upper bound	2(*L_N_*–*L*_1_)	d.f.
L5	1.12239	1.17804	1.23538	165.239	27
	1.12754		1.24098		
J3	1.11596	1.17407	1.23404	105.218	26
	1.12171		1.24032		
J6	1.15263	1.21385	1.27704	178.18	25
	1.15774		1.28258		
U6	1.17975	1.23815	1.29829	270.763	32
	1.18389		1.30274		
L4	1.17469	1.24032	1.30817	222.664	29
	1.17986		1.31378		
U5	1.18476	1.25169	1.32089	195.916	24
	1.18987		1.32643		
K6	1.18436	1.24819	1.31408	294.437	40
	1.18908		1.31919		
L1	1.28997	1.36794	1.44861	332.781	40
	1.29451		1.45349		
c4	1.08125	1.20363	1.33427	0.958866	9
	1.10312		1.36155		
b3	1.1223	1.18418	1.24816	75.4321	27
	1.12849		1.255		

**Table 3:** See description of Table 1 but for the Venezuela data set.

locus	lower bound	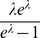	upper bound	2(*L_N_*–*L*_1_)	d.f.
J3	*N/A*	1	*N/A*	N/A	N/A
	*N/A*		*N/A*		
J6	*N/A*	1	*N/A*	N/A	N/A
	*N/A*		*N/A*		
U6	*N/A*	1	*N/A*	N/A	N/A
	*N/A*		*N/A*		
L4	*N/A*	1	*N/A*	N/A	N/A
	*N/A*		*N/A*		
U5	0.974273	1.02745	1.08251	8.32780	8
	1.00156		1.12327		
K6	0.971082	1.03104	1.09339	8.06610	3
	1.00176		1.13908		
L1	0.984526	1.04242	1.10251	0.00000	2
	1.00703		1.13188		
c4	0.973367	1.02863	1.08592	9.79400	3
	1.00163		1.1273		
b3	0.99278	1.06223	1.13479	3.66900	4
	1.01538		1.16345		
fr13	0.981231	1.05152	1.12504	0.20579	3
	1.00852		1.16137		
ps6	0.98346	1.04538	1.1098	0.00000	2
	1.00752		1.14139		
ps7	0.978848	1.02256	1.06754	1.01430	4
	1.00128		1.10032		

*N/A* indicates that that the method is not applicable (cf. Analysis, section 6).

From Figure 1 it is apparent that the estimates for MOI are highest in Kenya, followed by Cameroon, whereas they are very low in Venezuela. This is summarized in Figure 2 showing that the average ML estimates across the regions differ by several standard deviations.

The 95% profile-likelihood CIs for 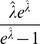, given by 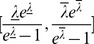, are reasonably large for the data sets from Cameroon and Venezuela (cf. Figure 1). However, due to the relatively small sample size, they are much less informative for the Kenya dataset.

The asymptotic confidence intervals agree well with the profile-likelihood CIs (cf. Figure 1 and Tables 1–3). This is particularly true for Cameroon, as expected because of the large sample size. The profile-likelihood CIs from the Kenya and Venezuela data are asymmetric while, the asymptotic CIs are - by definition - symmetric (however, the transformation 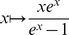 results in some asymmetry). (Note that, unlike profile-likelihood-based intervals, asymptotic CIs are not transformation respecting, i.e., 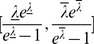 is the transformed CI of 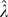, not the CI of 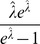.) In relative terms, this is more pronounced in Venezuela than in the Kenya data set. The reason is that the ML estimates 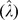 from the Venezuela data are close to zero, i.e., the boundary of the parameter range. This results in a very skewed likelihood function, yielding quite asymmetric profile-likelihood CIs. On the contrary, in Kenya, the ML estimates are rather large, and the likelihood function tends to be symmetric around its maximum.

Furthermore, we tested for pairwise differences between the estimates based on different marker loci. Tables 4–6 report the p-values for the likelihood-ratio, the Score, and the Wald test for the three regions. In all data sets, all tests perform equally well. There are some discrepancies, mainly due to the above mentioned skewness of the likelihood function. In the case of a skewed likelihood function, the likelihood-ratio test is the most preferable, because it accounts for the skewness.

**Table 4:** Pairwise tests of ML estimates from obtained from the Kenya data set.

locus	U7	L5	J3	J6	U6	L4	U5	K6	L1	c4	b3
U7	1.0000	**0.0000**	**0.0000**	**0.0000**	**0.0000**	**0.0000**	**0.0000**	**0.0000**	**0.0000**	**0.0000**	**0.0023**
	1.0000	**0.0006**	**0.0030**	**0.0056**	**0.0033**	**0.0012**	**0.0020**	**0.0000**	**0.0000**	**0.0048**	0.0514
	1.0000	**0.0004**	**0.0021**	**0.0043**	**0.0024**	**0.0007**	**0.0014**	**0.0000**	**0.0000**	**0.0034**	**0.0477**
L5	**0.0001**	1.0000	0.5316	0.2527	0.3527	0.6536	0.4511	0.2585	0.0866	0.4667	**0.0194**
	**0.0000**	1.0000	0.5097	0.2074	0.3159	0.6421	0.4236	0.2946	0.1258	0.4386	**0.0032**
	**0.0000**	1.0000	0.5092	0.2053	0.3145	0.6419	0.4228	0.2934	0.1237	0.4376	**0.0024**
J3	**0.0006**	0.5062	1.0000	0.6079	0.7762	0.8277	0.9252	0.0632	**0.0151**	0.9045	0.0790
	**0.0000**	0.5284	1.0000	0.5910	0.7708	0.8306	0.9246	0.1023	**0.0396**	0.9034	**0.0342**
	**0.0000**	0.5279	1.0000	0.5907	0.7707	0.8306	0.9246	0.1002	**0.0375**	0.9034	**0.0315**
J6	**0.0021**	0.2273	0.6101	1.0000	0.8096	0.4465	0.6574	**0.0141**	**0.0026**	0.7079	0.1987
	**0.0000**	0.2744	0.6265	1.0000	0.8136	0.4753	0.6696	**0.0394**	**0.0146**	0.7176	0.1378
	**0.0000**	0.2727	0.6262	1.0000	0.8136	0.4747	0.6694	**0.0374**	**0.0131**	0.7174	0.1347
U6	**0.0012**	0.3379	0.7826	0.8139	1.0000	0.6088	0.8439	**0.0292**	**0.0060**	0.8825	0.1333
	**0.0000**	0.3753	0.7878	0.8100	1.0000	0.6238	0.8464	0.0615	**0.0232**	0.8841	0.0769
	**0.0000**	0.3742	0.7878	0.8099	1.0000	0.6236	0.8464	0.0593	**0.0214**	0.8841	0.0736
L4	**0.0003**	0.6545	0.8381	0.4722	0.6207	1.0000	0.7571	0.1053	**0.0281**	0.7502	0.0516
	**0.0000**	0.6657	0.8353	0.4436	0.6058	1.0000	0.7510	0.1471	0.0583	0.7432	**0.0172**
	**0.0000**	0.6655	0.8353	0.4428	0.6055	1.0000	0.7510	0.1451	0.0561	0.7432	**0.0151**
U5	**0.0007**	0.4476	0.9290	0.6720	0.8474	0.7546	1.0000	**0.0498**	**0.0113**	0.9732	0.0941
	**0.0000**	0.4749	0.9296	0.6600	0.8448	0.7606	1.0000	0.0870	**0.0333**	0.9731	**0.0451**
	**0.0000**	0.4742	0.9296	0.6598	0.8448	0.7605	1.0000	0.0848	**0.0313**	0.9731	**0.0421**
K6	**0.0000**	0.3001	0.1091	**0.0331**	0.0526	0.1364	0.0744	1.0000	0.5466	0.0931	**0.0011**
	**0.0000**	0.2651	0.0698	**0.0120**	**0.0250**	0.0977	**0.0421**	1.0000	0.5613	0.0557	**0.0000**
	**0.0000**	0.2636	0.0674	**0.0105**	**0.0231**	0.0954	**0.0400**	1.0000	0.5610	0.0528	**0.0000**
L1	**0.0000**	0.1093	**0.0327**	**0.0075**	**0.0127**	**0.0396**	**0.0189**	0.5391	1.0000	**0.0278**	**0.0002**
	**0.0000**	0.0747	**0.0125**	**0.0012**	**0.0030**	**0.0180**	**0.0057**	0.5241	1.0000	**0.0097**	**0.0000**
	**0.0000**	0.0725	**0.0111**	**0.0008**	**0.0024**	**0.0165**	**0.0049**	0.5237	1.0000	**0.0083**	**0.0000**
c4	**0.0008**	0.4259	0.9016	0.6976	0.8754	0.7267	0.9709	**0.0453**	**0.0101**	1.0000	0.1006
	**0.0000**	0.4551	0.9027	0.6873	0.8737	0.7342	0.9710	0.0816	**0.0312**	1.0000	0.0500
	**0.0000**	0.4544	0.9027	0.6871	0.8736	0.7341	0.9710	0.0794	**0.0292**	1.0000	**0.0470**
b3	**0.0346**	**0.0067**	0.0552	0.1581	0.0916	**0.0237**	0.0541	**0.0000**	**0.0000**	0.0824	1.0000
	**0.0005**	**0.0333**	0.1130	0.2219	0.1529	0.0659	0.1086	**0.0026**	**0.0010**	0.1464	1.0000
	**0.0002**	**0.0308**	0.1099	0.2196	0.1501	0.0631	0.1057	**0.0021**	**0.0007**	0.1429	1.0000

The ML estimate obtained from the locus specified in the rows (*H*_0_) is tested against the estimates from the loci specified in the columns (*H_A_*). In each cell, the p-values for the likelihood-ratio (top), Score (middle), and Wald test (bottom) are shown. The Score and Wald tests are the version of eqs. (32) and (35), respectively. Significant differences are indicated in bold.

**Table 5:** See description of Table 4 but for the Cameroon data set.

locus	L5	J3	J6	U6	L4	U5	K6	L1	c4	b3
L5	1.0000	0.8960	0.2296	**0.0282**	**0.0428**	**0.0171**	**0.0176**	**0.0000**	0.6775	0.8465
	1.0000	0.8953	0.2552	**0.0439**	0.0637	**0.0312**	**0.0314**	**0.0000**	0.6899	0.8480
	1.0000	0.8953	0.2548	**0.0436**	0.0631	**0.0307**	**0.0310**	**0.0000**	0.6899	0.8480
J3	0.8896	1.0000	0.1787	**0.0184**	**0.0299**	**0.0113**	**0.0115**	**0.0000**	0.6282	0.7480
	0.8904	1.0000	0.2058	**0.0316**	**0.0483**	**0.0230**	**0.0229**	**0.0000**	0.6445	0.7522
	0.8904	1.0000	0.2054	**0.0313**	**0.0478**	**0.0226**	**0.0225**	**0.0000**	0.6445	0.7522
J6	0.2442	0.2195	1.0000	0.4042	0.4182	0.2490	0.2746	**0.0000**	0.8764	0.3811
	0.2189	0.1918	1.0000	0.4189	0.4340	0.2717	0.2956	**0.0001**	0.8746	0.3595
	0.2185	0.1913	1.0000	0.4188	0.4338	0.2713	0.2953	**0.0001**	0.8746	0.3593
U6	0.0601	0.0572	0.4610	1.0000	0.9489	0.6909	0.7583	**0.0002**	0.6138	0.1255
	**0.0406**	**0.0370**	0.4468	1.0000	0.9490	0.6956	0.7611	**0.0010**	0.5961	0.0979
	**0.0401**	**0.0364**	0.4467	1.0000	0.9490	0.6956	0.7611	**0.0010**	0.5961	0.0974
L4	0.0522	0.0500	0.4234	0.9428	1.0000	0.7394	0.8101	**0.0003**	0.5929	0.1122
	**0.0340**	**0.0311**	0.4075	0.9427	1.0000	0.7427	0.8119	**0.0012**	0.5734	0.0853
	**0.0335**	**0.0306**	0.4073	0.9427	1.0000	0.7427	0.8119	**0.0012**	0.5733	0.0848
U5	**0.0240**	**0.0240**	0.2604	0.6605	0.7426	1.0000	0.9160	**0.0011**	0.4914	0.0604
	**0.0125**	**0.0119**	0.2379	0.6554	0.7392	1.0000	0.9157	**0.0033**	0.4621	**0.0392**
	**0.0122**	**0.0116**	0.2376	0.6553	0.7392	1.0000	0.9157	**0.0032**	0.4620	**0.0388**
K6	**0.0307**	**0.0303**	0.3047	0.7436	0.8194	0.9192	1.0000	**0.0007**	0.5213	0.0736
	**0.0172**	**0.0162**	0.2838	0.7406	0.8178	0.9195	1.0000	**0.0025**	0.4950	0.0503
	**0.0169**	**0.0158**	0.2835	0.7406	0.8178	0.9195	1.0000	**0.0024**	0.4950	**0.0499**
L1	**0.0000**	**0.0000**	**0.0001**	**0.0002**	**0.0013**	**0.0035**	**0.0017**	1.0000	**0.0429**	**0.0000**
	**0.0000**	**0.0000**	**0.0000**	**0.0000**	**0.0003**	**0.0012**	**0.0005**	1.0000	**0.0145**	**0.0000**
	**0.0000**	**0.0000**	**0.0000**	**0.0000**	**0.0003**	**0.0011**	**0.0004**	1.0000	**0.0144**	**0.0000**
c4	0.3971	0.3531	0.7433	0.2282	0.2539	0.1366	0.1495	**0.0000**	1.0000	0.5589
	0.3781	0.3306	0.7469	0.2498	0.2771	0.1618	0.1738	**0.0000**	1.0000	0.5469
	0.3779	0.3303	0.7468	0.2495	0.2768	0.1613	0.1734	**0.0000**	1.0000	0.5468
b3	0.8332	0.7420	0.3252	0.0514	0.0710	**0.0306**	**0.0323**	**0.0000**	0.7548	1.0000
	0.8315	0.7378	0.3465	0.0708	0.0950	**0.0485**	**0.0498**	**0.0000**	0.7621	1.0000
	0.8315	0.7378	0.3463	0.0704	0.0945	**0.0480**	**0.0494**	**0.0000**	0.7621	1.0000

**Table 6:** See description of Table 4 but for the Venezuela data set.

locus	L4	U5	K6	L1	c4	b3	fr13	ps6	ps7
L4	*N/A*	*N/A*	*N/A*	*N/A*	*N/A*	*N/A*	*N/A*	*N/A*	*N/A*
	*N/A*	*N/A*	*N/A*	*N/A*	*N/A*	*N/A*	*N/A*	*N/A*	*N/A*
	*N/A*	*N/A*	*N/A*	*N/A*	*N/A*	*N/A*	*N/A*	*N/A*	*N/A*
U5	*N/A*	1.0000	0.9047	0.5670	0.9669	0.2143	0.4224	0.5133	0.8397
	*N/A*	1.0000	0.9084	0.6177	0.9673	0.3334	0.5093	0.5765	0.8291
	*N/A*	1.0000	0.9084	0.6173	0.9673	0.3317	0.5085	0.5759	0.8291
K6	*N/A*	0.9008	1.0000	0.6754	0.9349	0.2827	0.5108	0.6147	0.7371
	*N/A*	0.8968	1.0000	0.7045	0.9331	0.3859	0.5747	0.6553	0.7088
	*N/A*	0.8967	1.0000	0.7043	0.9331	0.3846	0.5741	0.6550	0.7086
L1	*N/A*	0.6415	0.7433	1.0000	0.6751	0.5352	0.7913	0.9253	0.4825
	*N/A*	0.5898	0.7164	1.0000	0.6329	0.5825	0.8035	0.9269	0.3846
	*N/A*	0.5895	0.7162	1.0000	0.6325	0.5821	0.8035	0.9268	0.3831
c4	*N/A*	0.9665	0.9367	0.6027	1.0000	0.2359	0.4512	0.5465	0.8047
	*N/A*	0.9660	0.9384	0.6457	1.0000	0.3501	0.5303	0.6018	0.7890
	*N/A*	0.9660	0.9384	0.6453	1.0000	0.3485	0.5296	0.6014	0.7889
b3	*N/A*	0.3485	0.4354	0.5643	0.3751	1.0000	0.7844	0.6391	0.2254
	*N/A*	0.2148	0.3242	0.5138	0.2505	1.0000	0.7711	0.6033	0.0871
	*N/A*	0.2128	0.3221	0.5129	0.2468	1.0000	0.7710	0.6028	0.0833
fr13	*N/A*	0.4858	0.5827	0.7771	0.5167	0.7529	1.0000	0.8551	0.3411
	*N/A*	0.3880	0.5150	0.7631	0.4303	0.7668	1.0000	0.8491	0.2077
	*N/A*	0.3869	0.5142	0.7630	0.4286	0.7667	1.0000	0.8490	0.2047
ps6	*N/A*	0.5865	0.6872	0.9235	0.6193	0.6056	0.8612	1.0000	0.4314
	*N/A*	0.5191	0.6477	0.9218	0.5626	0.6402	0.8667	1.0000	0.3188
	*N/A*	0.5186	0.6473	0.9218	0.5618	0.6399	0.8667	1.0000	0.3168
ps7	*N/A*	0.8500	0.7629	0.4201	0.8192	0.1342	0.3064	0.3773	1.0000
	*N/A*	0.8592	0.7853	0.5076	0.8323	0.2697	0.4269	0.4769	1.0000
	*N/A*	0.8592	0.7852	0.5066	0.8323	0.2675	0.4256	0.4758	1.0000

*N/A* indicates that that the test is not applicable (cf. Analysis, section 6). Results for loci J3, J6, and U6 are not shown because the tests are also not applicable (as for locus L4).

Tables 7–9 compare the three versions of the Score test, while Tables 10–12 compare those for the Wald test. The results are fairly consistent. However, the versions given by eqs. 34, 37 and 36 of the Score and Wald tests, respectively tend to be most inconsistent with the other tests, especially the likelihood-ratio test. The reason is that these use the roughest approximations.

**Table 7:** The same as Table 4 but with the p-value of the three versions, according to eqs. 33 (top), 32 (middle), and 34 (bottom) of the Score test.

locus	U7	L5	J3	J6	U6	L4	U5	K6	L1	c4	b3
U7	1.0000	**0.0000**	**0.0000**	**0.0000**	**0.0000**	**0.0000**	**0.0000**	**0.0000**	**0.0000**	**0.0000**	**0.0000**
	1.0000	**0.0006**	**0.0030**	**0.0056**	**0.0033**	**0.0012**	**0.0020**	**0.0000**	**0.0000**	**0.0048**	0.0514
	1.0000	**0.0000**	**0.0000**	**0.0000**	**0.0000**	**0.0000**	**0.0000**	**0.0000**	**0.0000**	**0.0000**	**0.0000**
L5	**0.0021**	1.0000	0.5434	0.2794	0.3740	0.6603	0.4671	0.2351	0.0656	0.4817	**0.0384**
	**0.0000**	1.0000	0.5097	0.2074	0.3159	0.6421	0.4236	0.2946	0.1258	0.4386	**0.0032**
	0.3113	1.0000	0.5732	0.3465	0.4250	0.6755	0.5040	0.1888	**0.0316**	0.5206	0.1439
J3	**0.0063**	0.4923	1.0000	0.6173	0.7793	0.8258	0.9255	**0.0429**	**0.0068**	0.9050	0.1112
	**0.0000**	0.5284	1.0000	0.5910	0.7708	0.8306	0.9246	0.1023	**0.0396**	0.9034	**0.0342**
	0.3298	0.4597	1.0000	0.6398	0.7867	0.8215	0.9264	**0.0159**	**0.0007**	0.9065	0.2261
J6	**0.0138**	0.1988	0.6004	1.0000	0.8072	0.4283	0.6498	**0.0057**	**0.0005**	0.7023	0.2335
	**0.0000**	0.2744	0.6265	1.0000	0.8136	0.4753	0.6696	**0.0394**	**0.0146**	0.7176	0.1378
	0.3467	0.1398	0.5755	1.0000	0.8014	0.3862	0.6317	**0.0004**	**0.0000**	0.6876	0.3336
U6	**0.0097**	0.3146	0.7796	0.8162	1.0000	0.5994	0.8423	**0.0156**	**0.0018**	0.8816	0.1685
	**0.0000**	0.3753	0.7878	0.8100	1.0000	0.6238	0.8464	0.0615	**0.0232**	0.8841	0.0769
	0.3387	0.2623	0.7718	0.8217	1.0000	0.5772	0.8386	**0.0029**	**0.0000**	0.8793	0.2785
L4	**0.0045**	0.6475	0.8397	0.4882	0.6292	1.0000	0.7607	0.0812	**0.0157**	0.7539	0.0801
	**0.0000**	0.6657	0.8353	0.4436	0.6058	1.0000	0.7510	0.1471	0.0583	0.7432	**0.0172**
	0.3235	0.6311	0.8437	0.5265	0.6492	1.0000	0.7688	**0.0428**	**0.0030**	0.7635	0.1944
U5	**0.0073**	0.4304	0.9287	0.6788	0.8488	0.7508	1.0000	**0.0315**	**0.0045**	0.9733	0.1276
	**0.0000**	0.4749	0.9296	0.6600	0.8448	0.7606	1.0000	0.0870	**0.0333**	0.9731	**0.0451**
	0.3326	0.3907	0.9279	0.6949	0.8523	0.7421	1.0000	**0.0096**	**0.0003**	0.9734	0.2417
K6	**0.0003**	0.3211	0.1338	0.0524	0.0747	0.1621	0.0985	1.0000	0.5373	0.1174	**0.0052**
	**0.0000**	0.2651	0.0698	**0.0120**	**0.0250**	0.0977	**0.0421**	1.0000	0.5613	0.0557	**0.0000**
	0.2855	0.3706	0.2116	0.1276	0.1488	0.2293	0.1703	1.0000	0.5161	0.1981	0.0752
L1	**0.0001**	0.1329	**0.0494**	**0.0169**	**0.0245**	0.0583	**0.0330**	0.5486	1.0000	**0.0435**	**0.0015**
	**0.0000**	0.0747	**0.0125**	**0.0012**	**0.0030**	**0.0180**	**0.0057**	0.5241	1.0000	**0.0097**	**0.0000**
	0.2725	0.1973	0.1205	0.0744	0.0833	0.1215	0.0923	0.5682	1.0000	0.1153	0.0541
c4	**0.0077**	0.4075	0.9010	0.7034	0.8764	0.7221	0.9709	**0.0279**	**0.0039**	1.0000	0.1345
	**0.0000**	0.4551	0.9027	0.6873	0.8737	0.7342	0.9710	0.0816	**0.0312**	1.0000	0.0500
	0.3337	0.3652	0.8994	0.7172	0.8787	0.7112	0.9707	**0.0078**	**0.0002**	1.0000	0.2480
b3	0.0808	**0.0015**	**0.0309**	0.1226	0.0610	**0.0095**	**0.0303**	**0.0000**	**0.0000**	0.0527	1.0000
	**0.0005**	**0.0333**	0.1130	0.2219	0.1529	0.0659	0.1086	**0.0026**	**0.0010**	0.1464	1.0000
	0.4077	**0.0000**	**0.0044**	0.0572	**0.0174**	**0.0005**	**0.0049**	**0.0000**	**0.0000**	**0.0119**	1.0000

**Table 8:** See description of Table 7 but for the Cameroon data set.

locus	L5	J3	J6	U6	L4	U5	K6	L1	c4	b3
L5	1.0000	0.8964	0.2148	**0.0208**	**0.0326**	**0.0111**	**0.0117**	**0.0000**	0.6733	0.8457
	1.0000	0.8953	0.2552	**0.0439**	0.0637	**0.0312**	**0.0314**	**0.0000**	0.6899	0.8480
	1.0000	0.8974	0.1800	**0.0090**	**0.0153**	**0.0033**	**0.0038**	**0.0000**	0.6509	0.8433
J3	0.8892	1.0000	0.1633	**0.0126**	**0.0214**	**0.0068**	**0.0071**	**0.0000**	0.6227	0.7460
	0.8904	1.0000	0.2058	**0.0316**	**0.0483**	**0.0230**	**0.0229**	**0.0000**	0.6445	0.7522
	0.8881	1.0000	0.1281	**0.0044**	**0.0084**	**0.0016**	**0.0018**	**0.0000**	0.5930	0.7395
J6	0.2585	0.2345	1.0000	0.3954	0.4087	0.2356	0.2621	**0.0000**	0.8770	0.3907
	0.2189	0.1918	1.0000	0.4189	0.4340	0.2717	0.2956	**0.0001**	0.8746	0.3595
	0.2959	0.2768	1.0000	0.3742	0.3858	0.2045	0.2330	**0.0000**	0.8801	0.4231
U6	0.0728	0.0699	0.4690	1.0000	0.9488	0.6881	0.7566	**0.0001**	0.6183	0.1385
	**0.0406**	**0.0370**	0.4468	1.0000	0.9490	0.6956	0.7611	**0.0010**	0.5961	0.0979
	0.1126	0.1132	0.4887	1.0000	0.9486	0.6814	0.7526	**0.0000**	0.6468	0.1884
L4	0.0643	0.0621	0.4324	0.9429	1.0000	0.7374	0.8091	**0.0001**	0.5979	0.1250
	**0.0340**	**0.0311**	0.4075	0.9427	1.0000	0.7427	0.8119	**0.0012**	0.5734	0.0853
	0.1030	0.1044	0.4545	0.9431	1.0000	0.7326	0.8066	**0.0000**	0.6292	0.1749
U5	**0.0327**	**0.0328**	0.2732	0.6635	0.7445	1.0000	0.9162	**0.0005**	0.4985	0.0713
	**0.0125**	**0.0119**	0.2379	0.6554	0.7392	1.0000	0.9157	**0.0033**	0.4621	**0.0392**
	0.0648	0.0684	0.3058	0.6706	0.7491	1.0000	0.9167	**0.0000**	0.5457	0.1184
K6	**0.0404**	**0.0401**	0.3166	0.7453	0.8204	0.9190	1.0000	**0.0003**	0.5278	0.0851
	**0.0172**	**0.0162**	0.2838	0.7406	0.8178	0.9195	1.0000	**0.0025**	0.4950	0.0503
	0.0747	0.0779	0.3465	0.7494	0.8227	0.9185	1.0000	**0.0000**	0.5701	0.1336
L1	**0.0000**	**0.0000**	**0.0003**	**0.0006**	**0.0027**	**0.0061**	**0.0033**	1.0000	**0.0493**	**0.0000**
	**0.0000**	**0.0000**	**0.0000**	**0.0000**	**0.0003**	**0.0012**	**0.0005**	1.0000	**0.0145**	**0.0000**
	**0.0008**	**0.0013**	**0.0033**	**0.0040**	**0.0113**	**0.0182**	**0.0118**	1.0000	0.1551	**0.0029**
c4	0.4075	0.3650	0.7412	0.2155	0.2402	0.1223	0.1355	**0.0000**	1.0000	0.5643
	0.3781	0.3306	0.7469	0.2498	0.2771	0.1618	0.1738	**0.0000**	1.0000	0.5469
	0.4341	0.3975	0.7360	0.1862	0.2082	0.0916	0.1051	**0.0000**	1.0000	0.5822
b3	0.8341	0.7443	0.3126	**0.0414**	0.0584	**0.0223**	**0.0239**	**0.0000**	0.7525	1.0000
	0.8315	0.7378	0.3465	0.0708	0.0950	**0.0485**	**0.0498**	**0.0000**	0.7621	1.0000
	0.8365	0.7503	0.2821	**0.0231**	**0.0343**	**0.0092**	**0.0105**	**0.0000**	0.7395	1.0000

**Table 9:** See descriptions of Table 7 but for the Venezuela data set and Table 6.

locus	L4	U5	K6	L1	c4	b3	fr13	ps6	ps7
L4	*N/A*	*N/A*	*N/A*	*N/A*	*N/A*	*N/A*	*N/A*	*N/A*	*N/A*
	*N/A*	*N/A*	*N/A*	*N/A*	*N/A*	*N/A*	*N/A*	*N/A*	*N/A*
	*N/A*	*N/A*	*N/A*	*N/A*	*N/A*	*N/A*	*N/A*	*N/A*	*N/A*
U5	*N/A*	1.0000	0.9028	0.5369	0.9666	0.1491	0.3702	0.4754	0.8446
	*N/A*	1.0000	0.9084	0.6177	0.9673	0.3334	0.5093	0.5765	0.8291
	*N/A*	1.0000	0.8967	0.4454	0.9659	**0.0316**	0.2222	0.3617	0.8587
K6	*N/A*	0.9027	1.0000	0.6587	0.9357	0.2236	0.4731	0.5912	0.7492
	*N/A*	0.8968	1.0000	0.7045	0.9331	0.3859	0.5747	0.6553	0.7088
	*N/A*	0.9085	1.0000	0.6072	0.9382	0.0878	0.3576	0.5181	0.7847
L1	*N/A*	0.6621	0.7546	1.0000	0.6927	0.5082	0.7847	0.9244	0.5216
	*N/A*	0.5898	0.7164	1.0000	0.6329	0.5825	0.8035	0.9269	0.3846
	*N/A*	0.7249	0.7888	1.0000	0.7443	0.4257	0.7639	0.9219	0.6381
c4	*N/A*	0.9667	0.9359	0.5774	1.0000	0.1722	0.4038	0.5137	0.8117
	*N/A*	0.9660	0.9384	0.6457	1.0000	0.3501	0.5303	0.6018	0.7890
	*N/A*	0.9674	0.9333	0.4999	1.0000	**0.0464**	0.2653	0.4136	0.8322
b3	*N/A*	0.4003	0.4779	0.5861	0.4258	1.0000	0.7902	0.6546	0.2899
	*N/A*	0.2148	0.3242	0.5138	0.2505	1.0000	0.7711	0.6033	0.0871
	*N/A*	0.5756	0.6147	0.6506	0.5849	1.0000	0.8083	0.7009	0.5189
fr13	*N/A*	0.5231	0.6091	0.7836	0.5514	0.7454	1.0000	0.8579	0.3961
	*N/A*	0.3880	0.5150	0.7631	0.4303	0.7668	1.0000	0.8491	0.2077
	*N/A*	0.6406	0.6907	0.8025	0.6546	0.7220	1.0000	0.8663	0.5710
ps6	*N/A*	0.6127	0.7032	0.9243	0.6426	0.5862	0.8584	1.0000	0.4764
	*N/A*	0.5191	0.6477	0.9218	0.5626	0.6402	0.8667	1.0000	0.3188
	*N/A*	0.6934	0.7522	0.9267	0.7109	0.5259	0.8494	1.0000	0.6131
ps7	*N/A*	0.8452	0.7502	0.3663	0.8119	0.0695	0.2346	0.3165	1.0000
	*N/A*	0.8592	0.7853	0.5076	0.8323	0.2697	0.4269	0.4769	1.0000
	*N/A*	0.8295	0.7092	0.2190	0.7891	**0.0029**	0.0744	0.1586	1.0000

**Table 10:** The same as Table 4 but with the p-value of the three versions, according to eqs. 35 (top), 37 (middle), and 36 (bottom) of the Wald test.

locus	U7	L5	J3	J6	U6	L4	U5	K6	L1	c4	b3
U7	1.0000	**0.0004**	**0.0021**	**0.0043**	**0.0024**	**0.0007**	**0.0014**	**0.0000**	**0.0000**	**0.0034**	**0.0477**
	1.0000	**0.0000**	**0.0000**	**0.0000**	**0.0000**	**0.0000**	**0.0000**	**0.0000**	**0.0000**	**0.0000**	**0.0000**
	1.0000	**0.0000**	**0.0000**	**0.0000**	**0.0000**	**0.0000**	**0.0000**	**0.0000**	**0.0000**	**0.0000**	**0.0000**
L5	**0.0000**	1.0000	0.5092	0.2053	0.3145	0.6419	0.4228	0.2934	0.1237	0.4376	**0.0024**
	**0.0019**	1.0000	0.5406	0.2703	0.3667	0.6579	0.4613	0.2448	0.0723	0.4784	**0.0319**
	0.1722	1.0000	0.5728	0.3442	0.4236	0.6753	0.5032	0.1877	**0.0306**	0.5197	0.1322
J3	**0.0000**	0.5279	1.0000	0.5907	0.7707	0.8306	0.9246	0.1002	**0.0375**	0.9034	**0.0315**
	**0.0057**	0.4969	1.0000	0.6144	0.7784	0.8264	0.9254	0.0503	**0.0089**	0.9049	0.1020
	0.2203	0.4592	1.0000	0.6395	0.7866	0.8215	0.9264	**0.0152**	**0.0006**	0.9065	0.2188
J6	**0.0000**	0.2727	0.6262	1.0000	0.8136	0.4747	0.6694	**0.0374**	**0.0131**	0.7174	0.1347
	**0.0126**	0.2075	0.6026	1.0000	0.8079	0.4341	0.6522	**0.0082**	**0.0009**	0.7034	0.2247
	0.2609	0.1384	0.5752	1.0000	0.8013	0.3855	0.6315	**0.0004**	**0.0000**	0.6875	0.3298
U6	**0.0000**	0.3742	0.7878	0.8099	1.0000	0.6236	0.8464	0.0593	**0.0214**	0.8841	0.0736
	**0.0088**	0.3220	0.7803	0.8155	1.0000	0.6025	0.8428	**0.0201**	**0.0027**	0.8818	0.1591
	0.2422	0.2612	0.7718	0.8216	1.0000	0.5769	0.8385	**0.0027**	**0.0000**	0.8793	0.2731
L4	**0.0000**	0.6655	0.8353	0.4428	0.6055	1.0000	0.7510	0.1451	0.0561	0.7432	**0.0151**
	**0.0040**	0.6499	0.8393	0.4831	0.6265	1.0000	0.7594	0.0905	**0.0192**	0.7531	0.0715
	0.2043	0.6309	0.8437	0.5257	0.6489	1.0000	0.7688	**0.0418**	**0.0028**	0.7634	0.1857
U5	**0.0000**	0.4742	0.9296	0.6598	0.8448	0.7605	1.0000	0.0848	**0.0313**	0.9731	**0.0421**
	**0.0065**	0.4360	0.9288	0.6767	0.8484	0.7521	1.0000	**0.0380**	**0.0062**	0.9733	0.1183
	0.2273	0.3900	0.9279	0.6947	0.8523	0.7420	1.0000	**0.0091**	**0.0003**	0.9734	0.2350
K6	**0.0000**	0.2636	0.0674	**0.0105**	**0.0231**	0.0954	**0.0400**	1.0000	0.5610	0.0528	**0.0000**
	**0.0003**	0.3131	0.1275	**0.0445**	0.0658	0.1517	0.0883	1.0000	0.5406	0.1116	**0.0033**
	0.1060	0.3691	0.2076	0.1210	0.1433	0.2262	0.1659	1.0000	0.5158	0.1929	0.0585
L1	**0.0000**	0.0725	**0.0111**	**0.0008**	**0.0024**	**0.0165**	**0.0049**	0.5237	1.0000	**0.0083**	**0.0000**
	**0.0001**	0.1233	**0.0449**	**0.0126**	**0.0192**	0.0500	**0.0264**	0.5441	1.0000	**0.0395**	**0.0008**
	0.0769	0.1939	0.1147	0.0665	0.0764	0.1166	0.0864	0.5679	1.0000	0.1080	**0.0364**
c4	**0.0000**	0.4544	0.9027	0.6871	0.8736	0.7341	0.9710	0.0794	**0.0292**	1.0000	**0.0470**
	**0.0069**	0.4135	0.9011	0.7016	0.8760	0.7236	0.9709	**0.0339**	**0.0054**	1.0000	0.1252
	0.2301	0.3644	0.8994	0.7171	0.8787	0.7111	0.9707	**0.0074**	**0.0002**	1.0000	0.2416
b3	**0.0002**	**0.0308**	0.1099	0.2196	0.1501	0.0631	0.1057	**0.0021**	**0.0007**	0.1429	1.0000
	0.0779	**0.0022**	**0.0348**	0.1306	0.0677	**0.0123**	**0.0355**	**0.0000**	**0.0000**	0.0571	1.0000
	0.3745	**0.0000**	**0.0040**	0.0560	**0.0166**	**0.0004**	**0.0045**	**0.0000**	**0.0000**	**0.0112**	1.0000

**Table 11:** See description of Table 10 but for the Cameroon data set.

locus	L5	J3	J6	U6	L4	U5	K6	L1	c4	b3
L5	1.0000	0.8953	0.2548	**0.0436**	0.0631	**0.0307**	**0.0310**	**0.0000**	0.6899	0.8480
	1.0000	0.8963	0.2184	**0.0226**	**0.0350**	**0.0125**	**0.0131**	**0.0000**	0.6684	0.8456
	1.0000	0.8974	0.1797	**0.0089**	**0.0151**	**0.0033**	**0.0037**	**0.0000**	0.6509	0.8433
J3	0.8904	1.0000	0.2054	**0.0313**	**0.0478**	**0.0226**	**0.0225**	**0.0000**	0.6445	0.7522
	0.8893	1.0000	0.1669	**0.0140**	**0.0234**	**0.0078**	**0.0081**	**0.0000**	0.6163	0.7458
	0.8881	1.0000	0.1278	**0.0043**	**0.0083**	**0.0015**	**0.0017**	**0.0000**	0.5930	0.7395
J6	0.2185	0.1913	1.0000	0.4188	0.4338	0.2713	0.2953	**0.0001**	0.8746	0.3593
	0.2549	0.2320	1.0000	0.3979	0.4114	0.2393	0.2657	**0.0000**	0.8778	0.3923
	0.2954	0.2763	1.0000	0.3740	0.3857	0.2042	0.2328	**0.0000**	0.8801	0.4229
U6	**0.0401**	**0.0364**	0.4467	1.0000	0.9490	0.6956	0.7611	**0.0010**	0.5961	0.0974
	0.0694	0.0676	0.4668	1.0000	0.9488	0.6890	0.7571	**0.0001**	0.6259	0.1411
	0.1117	0.1122	0.4886	1.0000	0.9486	0.6813	0.7526	**0.0000**	0.6467	0.1878
L4	**0.0335**	**0.0306**	0.4073	0.9427	1.0000	0.7427	0.8119	**0.0012**	0.5733	0.0848
	0.0610	0.0599	0.4299	0.9429	1.0000	0.7380	0.8094	**0.0001**	0.6062	0.1275
	0.1021	0.1034	0.4543	0.9431	1.0000	0.7326	0.8066	**0.0000**	0.6292	0.1743
U5	**0.0122**	**0.0116**	0.2376	0.6553	0.7392	1.0000	0.9157	**0.0032**	0.4620	**0.0388**
	**0.0302**	**0.0311**	0.2696	0.6626	0.7439	1.0000	0.9162	**0.0006**	0.5114	0.0736
	0.0638	0.0673	0.3054	0.6705	0.7491	1.0000	0.9167	**0.0000**	0.5456	0.1177
K6	**0.0169**	**0.0158**	0.2835	0.7406	0.8178	0.9195	1.0000	**0.0024**	0.4950	**0.0499**
	**0.0377**	**0.0382**	0.3133	0.7448	0.8201	0.9191	1.0000	**0.0004**	0.5393	0.0876
	0.0738	0.0768	0.3462	0.7494	0.8227	0.9185	1.0000	**0.0000**	0.5700	0.1329
L1	**0.0000**	**0.0000**	**0.0000**	**0.0000**	**0.0003**	**0.0011**	**0.0004**	1.0000	**0.0144**	**0.0000**
	**0.0000**	**0.0000**	**0.0002**	**0.0005**	**0.0021**	**0.0050**	**0.0026**	1.0000	0.0769	**0.0001**
	**0.0006**	**0.0010**	**0.0029**	**0.0038**	**0.0108**	**0.0176**	**0.0113**	1.0000	0.1547	**0.0026**
c4	0.3779	0.3303	0.7468	0.2495	0.2768	0.1613	0.1734	**0.0000**	1.0000	0.5468
	0.4050	0.3631	0.7418	0.2191	0.2439	0.1262	0.1394	**0.0000**	1.0000	0.5652
	0.4339	0.3972	0.7360	0.1859	0.2079	0.0912	0.1048	**0.0000**	1.0000	0.5821
b3	0.8315	0.7378	0.3463	0.0704	0.0945	**0.0480**	**0.0494**	**0.0000**	0.7621	1.0000
	0.8339	0.7439	0.3157	**0.0439**	0.0615	**0.0243**	**0.0260**	**0.0000**	0.7496	1.0000
	0.8365	0.7503	0.2818	**0.0229**	**0.0340**	**0.0090**	**0.0104**	**0.0000**	0.7395	1.0000

**Table 12:** See description of Table 10 but for the Venezuela data set and Table 6.

locus	L4	U5	K6	L1	c4	b3	fr13	ps6	ps7
L4	*N/A*	*N/A*	*N/A*	*N/A*	*N/A*	*N/A*	*N/A*	*N/A*	*N/A*
	*N/A*	*N/A*	*N/A*	*N/A*	*N/A*	*N/A*	*N/A*	*N/A*	*N/A*
	*N/A*	*N/A*	*N/A*	*N/A*	*N/A*	*N/A*	*N/A*	*N/A*	*N/A*
U5	*N/A*	1.0000	0.9084	0.6173	0.9673	0.3317	0.5085	0.5759	0.8291
	*N/A*	1.0000	0.9026	0.5369	0.9666	0.1482	0.3678	0.4747	0.8446
	*N/A*	1.0000	0.8967	0.4449	0.9659	**0.0310**	0.2213	0.3610	0.8587
K6	*N/A*	0.8967	1.0000	0.7043	0.9331	0.3846	0.5741	0.6550	0.7086
	*N/A*	0.9028	1.0000	0.6587	0.9357	0.2227	0.4712	0.5907	0.7494
	*N/A*	0.9084	1.0000	0.6070	0.9382	0.0870	0.3569	0.5177	0.7845
L1	*N/A*	0.5895	0.7162	1.0000	0.6325	0.5821	0.8035	0.9268	0.3831
	*N/A*	0.6639	0.7555	1.0000	0.6931	0.5076	0.7843	0.9244	0.5221
	*N/A*	0.7246	0.7887	1.0000	0.7440	0.4252	0.7638	0.9219	0.6371
c4	*N/A*	0.9660	0.9384	0.6453	1.0000	0.3485	0.5296	0.6014	0.7889
	*N/A*	0.9667	0.9359	0.5774	1.0000	0.1713	0.4016	0.5131	0.8118
	*N/A*	0.9674	0.9333	0.4995	1.0000	**0.0457**	0.2645	0.4129	0.8322
b3	*N/A*	0.2128	0.3221	0.5129	0.2468	1.0000	0.7710	0.6028	0.0833
	*N/A*	0.4069	0.4826	0.5862	0.4271	1.0000	0.7907	0.6551	0.2912
	*N/A*	0.5740	0.6132	0.6499	0.5820	1.0000	0.8082	0.7005	0.5138
fr13	*N/A*	0.3869	0.5142	0.7630	0.4286	0.7667	1.0000	0.8490	0.2047
	*N/A*	0.5270	0.6117	0.7836	0.5521	0.7452	1.0000	0.8579	0.3970
	*N/A*	0.6399	0.6901	0.8024	0.6534	0.7219	1.0000	0.8662	0.5684
ps6	*N/A*	0.5186	0.6473	0.9218	0.5618	0.6399	0.8667	1.0000	0.3168
	*N/A*	0.6152	0.7046	0.9243	0.6430	0.5857	0.8582	1.0000	0.4771
	*N/A*	0.6930	0.7520	0.9267	0.7103	0.5256	0.8494	1.0000	0.6116
ps7	*N/A*	0.8592	0.7852	0.5066	0.8323	0.2675	0.4256	0.4758	1.0000
	*N/A*	0.8449	0.7495	0.3662	0.8118	0.0689	0.2318	0.3156	1.0000
	*N/A*	0.8295	0.7091	0.2180	0.7890	**0.0028**	0.0736	0.1576	1.0000

Overall, the methods perform well for all data sets and provide meaningful results. However, the statistical tests also yielded significant differences in some of the pairwise comparisons of the various 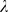 estimates in each region (Tables 4–12). The allele frequencies differ of course but all are based on the same true parameter 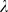. If the estimates for 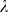 are significantly different, some of them cannot be trusted. This can have various reasons. First, it can be a type I error. However, this occurs only with small probability if the CIs are well calibrated, i.e., their nominal coverage (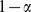) is close to the actual coverage. Asymptotic CIs and tests based on them (Wald, Score) will be more affected than profile-likelihood-based intervals, because the former are inherently forced to be symmetric. This is particularly true if the estimates for 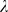 are close to zero. To quantify this effect, and to suggest heuristic methods to recalibrate the CIs, a systematic numerical robustness study of the approach is planned. Preliminary investigations, however, have shown that particularly the profile-likelihood-based CIs are well calibrated.

Second, the tests are designed to compare the ML estimate based on the data with a value 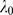, which has to be interpreted as prior knowledge. Strictly speaking, it is not meant to be estimated from data itself, or at least data which is available. A test designed to compare two estimates, should incorporate information from both data sets (data from both markers). A standard approach to resolve this is as follows. One could calculate the product of the maximum likelihood from both markers and compare it with the maximum likelihood of both markers conditioned on equality of 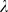. This however would require much more numerical effort than the tests here. Note further, that the structure of the data does not allow to perform a permutation test, because the allele-frequency distributions are expected to be different. This is true for two different marker loci in the same endemic region as well as for the same marker in two different populations.

Third, the model assumptions might be violated, i.e., the underlying Poisson distribution might not be correct. This can again be quantified in the coarse of a robustness study.

Fourth, the allele-frequency spectra of two different marker loci is very different, and the method might be sensitive to this. For instance strong skewness in the data distributions might bias the estimates. This is obviously the case if one marker shows no variation at all. Moreover, the number of different allele at different markers is very different, which results in very different probabilities of the ML estimates. These issues again need to be investigated in a numerical study.

Fifth, some STR markers tend to be hyper-mutable. As a result, not just the frequency distribution might be more problematic, but it is also more challenging to correctly identify the tandem repeat numbers. Hence, for hyper-mutable markers the data might have very bad quality. In our examples the marker labelled L1 appears to be hyper-mutable.

Because of all these possible reasons, it would be pre-mature to suggest a heuristic on how to decide, which estimates can be trusted the most. A systematic numerical follow-up study is planned to investigate all these possibilities in detail to provide suggestions on the criteria upon which the data is chosen.

## Discussion

The number of genetically distinct lineages co-infecting a host - commonly referred to as “multiplicity of infection” (MOI) - is a key quantity in epidemiology. First, it relates with transmission intensity since it provides a metric for the number of secondary infections after a primary infection; assuming that the lineages circulating are identifiable (e.g. secondary infections within a clonal outbreak simply cannot be traceable). Second, it measures the possibility of genetic exchange among those lineages as determined by the genetic system of the pathogen in question. Finally, if phenotypic differences are associated with those lineages, MOI could lead to very complex dynamics driven by natural selection.

Measuring MOI is desirable in a variety of infectious diseases, but - in many instances - only feasible if it can be measured at low cost and with a reasonable effort. Optimally it should fit into standard study designs and should be easily computable with whatever genotyping data can be collected from clinical specimens. In order to meet these goals, we further developed the maximum-likelihood (ML) method originally proposed by [20] and applied it to three malaria datasets as examples.

From a total of 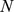 samples (e.g. blood samples), the number of genetically distinguishable lineages present in each host are recorded. From the resulting data, assuming that hosts are infected randomly by those lineages according to their prevalence, we derived the likelihood function. If infections with the pathogen are rare events, a natural choice for the number of co-infecting lineages is a conditional Poisson distribution (CPD). This distribution comes with the appealing feature that it is characterized by a single parameter 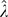, whose transform 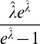 is the average MOI. Assuming a CPD, the likelihood function simplifies as well as the procedure to derive the ML estimates. Although, this was previously described by [20], we were able to derive a number of important results: First, the ML estimate always exists and is unique. Second, it has the intuitive interpretation of being the parameter vector under which the observed are the expected prevalences for the distinguishable lineages, i.e., the observation is the expectation, if the ML estimate is the true parameter vector. Third, the recursion to compute the ML estimate for 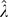 reduced from a multi- to a one-dimensional recursion, which just depends on the number of samples 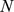 and the observed prevalences. The ML estimates for the lineages frequencies are explicit functions of 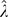. Fourth, the recursion for 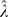 converges (at least) from every initial value 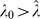. Convergence is monotonically, at quadratic rate, and typically occurs within a few iterations. Besides the obvious computational advantages provided of our results their actual foremost importance is that they justify the ML approach. Using an ML estimates is only appropriate if it has a significantly higher probability than distant alternative parameter choices, which is difficult to evaluate in a multi-dimensional space. However, the form of the ML estimate here - particularly because the lineages prevalences depend continuously on 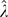 - indicates that the observation will have significantly lower probability under distant alternative parameter choices. The method worked well for the three malaria datasets to which it was applied, and gave similar results when applied to different independent microsatellite loci.

Although, our results justify the ML approach, it is nevertheless of fundamental importance to provide confidence intervals (CIs). We reported here on asymptotic and profile-likelihood-based CIs for all parameters. Asymptotic CIs are either based on the observed or the expected Fisher information, which under the CPD coincide. Explicit formulas for the CIs for all involved parameters were derived. Profile-likelihood based CIs were already emphasized by [20]. However, it was important to note that they can actually be derived at low numerical costs by using the method of Lagrange multiplies. This reduces the numerical effort to the same magnitude as for the ML estimate. Assuming the CPD, we proved that the CI for the parameter 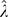, yielding the estimate for the MOI, is uniquely defined. The confidence bounds are derived by a two-dimension recursion, which converges locally at quadratic rate. Both kinds of CIs gave meaningful results for the three data sets to which we applied the methods and they agree well. Although the asymptotic CIs are easier to derive, we suggest to use the profile-likelihood-based CIs if sample size is low and/or the ML estimate for 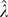 is small for the reasons discussed in the application section. Although, we discussed CIs for the linages' frequencies, these are somewhat less interesting, unless one focuses on the prevalence of a particular linage. Otherwise one should derive confidence regions on the simplex for the lineage frequencies, which is done as outlined, but numerically more demanding.

To test the ML estimate against other parameter choices typically three statistical tests are used, the likelihood-ratio, the Score, and the Wald test. The latter two are based on the asymptotic CIs, while the likelihood-ratio test builds upon the profile-likelihood-based CIs. Motivated by our intention to apply the methods to malaria we focused on using these tests to compare estimates for the parameter 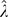. Namely, several genetic markers characterizing linages are typically available (e.g., several microsatellite markers), to all of which the methods are applicable. While the true parameter 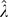 is of course the same for all markers, the ML estimates obtained from them will differ. It is therefore important to test whether these estimates differ significantly. The parameter 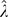 changes on temporal and spatial scales. An obvious question is, whether MOI changes over time (e.g. before and after the implementation of control measures) or varies across endemic regions. Hence, it is important to test for significant differences in estimates for 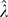.

Not surprisingly all tests described perform equally well as they are asymptotically equivalent. However, as in the case of CIs we suggest to use the likelihood-ratio test if sample size is small or the parameters compared are small. If interested in p-values additional effort is required for the likelihood-ratio test, because a two-dimensional iteration needs to be performed. However, numerically this is only as demanding as obtaining the CIs. Because the test statistics for the Score and Wald tests can be derived, it is easy to derive p-values in these cases. For each of these two tests we provided three alternative variants, which all worked almost equally well in the provided examples. We should point out that it was our intention to indicate only how tests for the parameters can be constructed. With the usual approaches one could compare multiple parameters at the same time, including the information of all these markers. This however, exceeds both our intention and the scope of this article. Finally, as a justification for using the CPD, which simplifies the method to a great extent, we summarized the test suggested by [20]. Although the test will be uninformative if many lineages are present it provides a justification for the approach. Of note, the CPD is an intuitive assumption if infections are relatively rare events. This does not relate with the overall prevalence but rather with how high the observed incidence is in a given population in terms of the time scale required for the pathogen to complete its transmission cycle. Such relationship is hard to establish without complex simulations but it is worth noting that there could be biologic scenarios (particular pathogens or epidemiologic settings) where this assumption does not hold. Thus, it is advisable to check whether the CPD assumption is violated using the tests for the model fit proposed in this investigation. In our case of study, we observe robust estimates across very different epidemiologic settings. Overall, the methods developed here can be used to compare groups under different exposures, different manifestations of disease, groups of patients that have different genotypes (e.g. sickle cell or any other hemoglobinopathies associated with protection), or the efficacy of a given vaccine. Biologically, this method assumes that the rate of evolution of the marker used is “low” relative to the time of the infection. That is, there is a “numerable” set of lineages that can be estimated and no variants are generated during the time scale of one infection. Thus, it is not suitable for pathogens such as HIV or any other hypervariable virus. The second assumption is that the set of markers used to detect and characterize the MOI are effectively neutral, so they are not linked to genes under selection. Thus, the loci cannot be associated with antigens or drug resistance. As presented, each loci is considered independent, which is a typical assumption of genotyping base approximations used in molecular epidemiology. We also want to emphasize that this MOI estimate depends on the number of detectable lineages given a laboratory method. Thus, results from different markers such SNPs or microsatellites are expected to differ as a function of their differences in mutation rates and mode of evolution. One could actually calculate the fit of individual loci and then exclude potential outliers if there is any biological reason to do so (e.g. microsatellites under different evolutionary models where one is hyper-variable or non-variable when compared with others). The method is sensitive enough to detect differences in MOI under different epidemiologic settings as indicated by the analyses of empirical data. Whereas this is not per se a “genomic” method, in the sense that is not designed to estimate MOI directly from reads generated from next generation sequence (NGS) data, it can do so from a given set of SNPs or microsatellites detected by using NGS. Whereas the method was originally intended for applications to malaria, it can be applied to other parasitic or microbial diseases where the assumptions are not violated. E.g. variation on the VNTRs in a multi-clonal infection of Mycobacterium tuberculosis. Unlike empirical approaches where simply alleles are counted and then averaged, the proposed ML method provides a robust and computationally efficient statistical framework that can be integrated in epidemiological investigations.

## Analysis

### 1 The Model

#### 1.1 Background

Here, 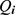 given by (1) is explicitly derived under the assumption that 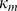 is given by the CPD (2). Namely, 
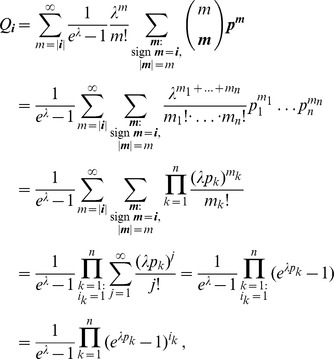
where in the derivations the condition 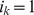 indicates that the product is taken over all non-zero components of 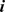, corresponding to the alleles found in a sample with allele configuration 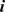.

#### 1.2 Log-Likelihood

Assuming that the number of lineages infecting a host follows the CPD (2), the log-likelihood (3) simplifies to 
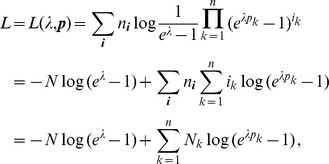
where



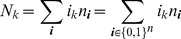
is the number of samples that contain allele 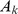. Notably, 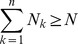 with equality only if all samples are single infections.

#### 1.3 Proof of Remark 1

The proof of Remark 1 is as follows.

**Proof of Remark 1.** First, note that 
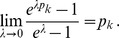



Moreover, using de l'Hospitals rule we see that 
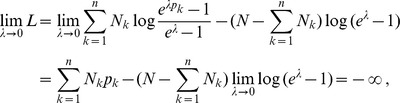
because 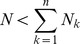 (note that this holds also true if 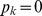 for some 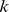). This proves that 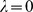 is not a maximum likelihood estimate, which is quite intuitive. 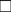


#### 1.4 Derivatives of the log-likelihood

Assuming the CPD (2) the log-likelihood function is given by (4) and the derivatives of (5) are hence straightforwardly calculated to be 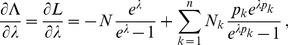
(39a)

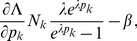
(39b)

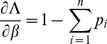
(39c)

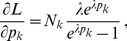
(39d)

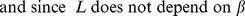


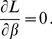
(39e)


The entries of the Hessian matrix (7), i.e., the second derivatives of 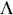, given by (5), are calculated to be 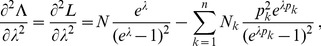
(40a)

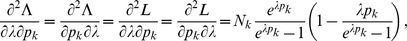
(40b)

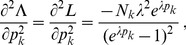
(40c)

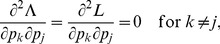
(40d)

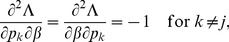
(40e)

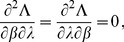
(40f)

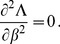
(40g)


### 2 Proofs of the main results

#### 2.1 Existence and uniqueness of the ML estimate

First, the result showing existence and uniqueness of the ML estimate in the generic case is proven.

**Proof of Result 1.** Assume 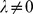, as this cannot be the ML estimate according to Remark 1. Equating (39b) to zero yields 
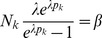
 for all 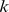. Substituting this into (39a) and setting the equation to zero yields 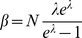. Therefore, we obtain 
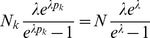
 or 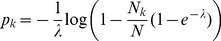
(41)proving the last assertion. Hence, it remains to prove the statements for 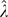.

By using (41) and equating (39c) to zero, we obtain 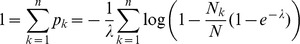, which is equivalent to 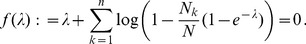
(42)Therefore, the ML estimate is a solution of (42). Straightforward calculation gives



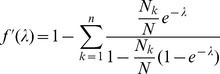
and



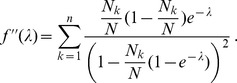



Note that, 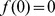 and 
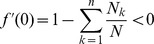
, because 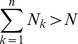. Hence, 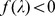 near zero. Note further that 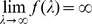. Hence, 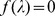 has at least one positive solution. Since, 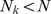 for at least one 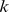, 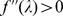, implying that 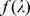 is strictly convex for 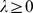. Because 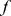 is strictly convex there can be at most one positive solution 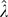 of 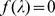. Moreover, 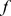 is strictly monotonically increasing for 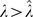.

The solution can be found by a Newton method. Because 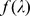 is strictly convex and monotonically increasing for 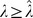, the Newton method converges monotonically to the solution 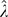. Moreover, because 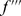 is continuous, the rate of convergence is at least quadratic. Noting that 
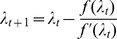
 yields (9) completes the proof.
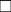


The special case, in which only single infections occur, is summarized by Remark 2. It can be proven as follows.

**Proof of Remark 2.** Examining the proof of Result 1 yields that that the ML estimate is any positive root of 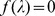. In the present case 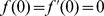. However, since 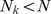 must hold for at least one 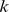, 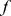 is still strictly convex. This implies that 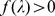 for all 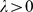. Hence, no maximum likelihood estimate with 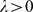 exists.

Moreover, since 
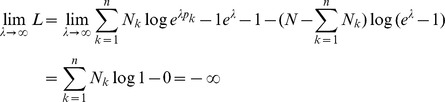
the ML estimate can only be attained at 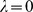.

In the limit 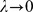, one obtains, as in the proof of Remark 1, 
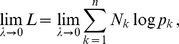
which is maximized at 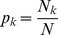. Particularly, the likelihood function is finite in this case. 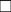


In the other non-generic situation, every lineage is found in all samples, which is described in Remark 3 and can be proven as follows.

**Proof of Remark 3.** The proof of Result 1 yields 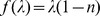. Hence, 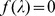 has no positive solution, and hence no ML estimate with 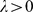 exists. Clearly, Remark 1 states that 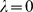 is also not an ML estimate.

In this case the log-likelihood function simplifies to 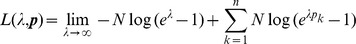


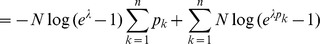


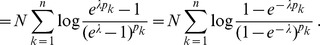



Taking the limit 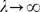 yields 
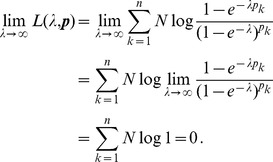



Since 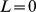 implies that the likelihood is one, this limit case, which is - of note - independent of the allele-frequency distribution, is the maximum likelihood.
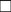


Remark 4 states that the expected number of samples containing a given lineage equals the observed number of samples containing this allele if the ML estimate is the true parameter. The proof is as follows.

**Proof of Remark 4.** The maximum likelihood estimate satisfies 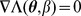. Equating (39b) to zero yields 
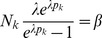
 for all 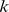. Substituting this into (39a) and setting the equation to zero yields 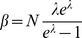. Therefore, we obtain 
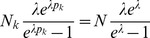
 or 
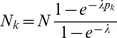
. Hence, it remains to be shown that 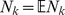 holds.

In the following we will use that 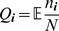. To simplify the notation assume 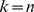. Hence, 
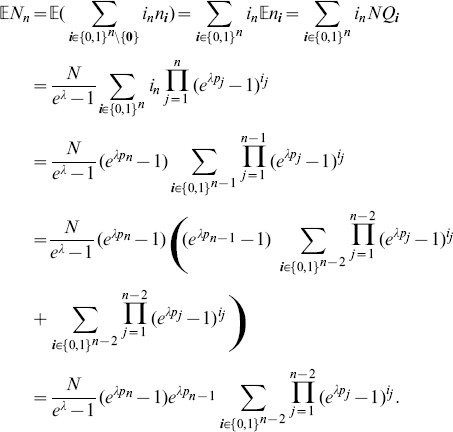



Successively repeating the last step gives 
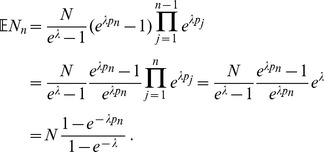



Since the alleles can be arbitrarily labeled, we obtain 
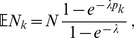
(43)


The proof is completed by noting that 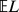 is obtained from (4) by replacing 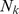 with 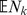.

#### 2.2 Profile likelihood based confidence intervals

The existence sand uniqueness of the profile-likelihood-based confidence intervals are proven as follows.

**Proof of Result 2.** The proof consists of several parts.

Part A: *Existence in the generic case.* We first assume 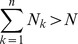 and 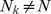 for at least one 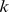 and prove the CI's existence.

The CI's bounds satisfy (12). The equations 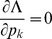, yield 
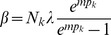
, or 
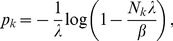
(44)which implies that 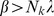 must hold for all 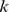. Since, 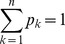, by summing up the above expression one arrives at 
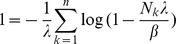
. Thus, for fixed 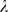 the Lagrange multiplier 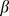 is a zero of the function



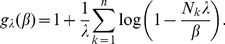
(45)Its derivative is given by 
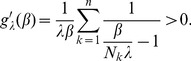
(46)


Hence, 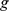 is strictly monotonically increasing in 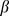, and consequently has at most one zero 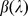. Note that 
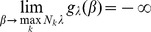
 and 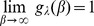. Hence, 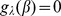 has exactly one solution 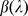. Furthermore, according to the implicit-function theorem, 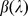 is a continuously differentiable function of 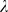.

The likelihood function (4) can be rewritten as 
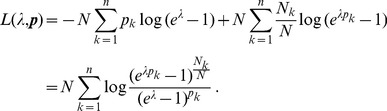
(47)


Note that 
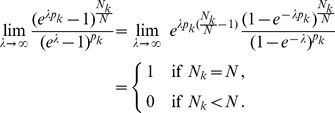



Since 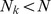 for at least one 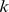, it follows that 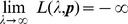
(48)for any arbitrary but fixed allele-frequency vector 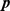. Moreover, the proof of Remark 1 reveals that



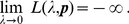
(49)Now, for any 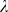, let 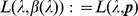 with 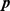 given by (44) with 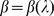.

Next, we show indirectly that 
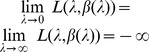
.

First, assume 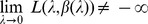. Hence, there exists a sequence 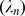, with 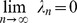 but 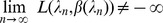. Hence, 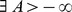 such that for a subsequence 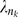, 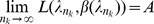. Without loss of generality, 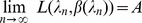. Let 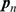 be the corresponding sequence of allele-frequency vectors. Since the simplex is compact, there exists a convergent subsequence 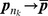. Because 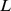 is continuous, it follows that 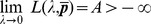, contradicting (48).

Analogously it is shown that 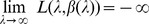.

Since 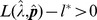, 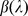 as well as 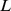 are continuous, and 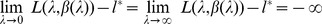, there exist 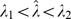, such that 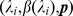 is a solution of (12), where 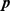 is given by (44). This proves the existence of the CI's bounds.

Part B: *Uniqueness in the generic case.* Next, the uniqueness of the confidence intervals is proven. Assume two values 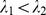 with 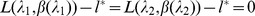. Since 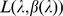 is continuously differentiable the mean value theorem implies that there exists 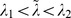 with 
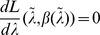
. Application of the chain rule yields 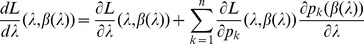. By definition of 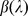, the relation 
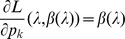
 holds. Hence, 
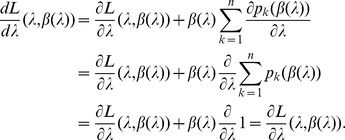
 Thus,


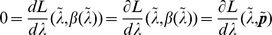
, where 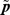 is given by (44) with 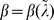. This implies that 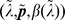 is a zero of (39), or, in other words, that 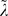 is a maximum likelihood estimate. Because of its uniqueness 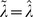, and 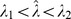. Hence, 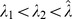 or 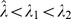 is impossible, and the CI is therefore uniquely defined.

Part C: *Existence and uniqueness in the non-generic cases.* In the case 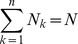 the same proof holds with obvious modifications. As (49) is violated and becomes 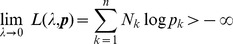. It follows that at least one solution of (12) exist. The above proof of uniqueness, implies that this is the only solution.

Similarly, for 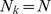 for all 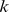, (48) is violated and becomes 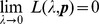, from which the existence of exactly one solution of (12) follows from the same proof as in the generic case.

Part D: *Derivation of the CIs in the generic case.* Parts A and B reveal that the bounds of the CI's bounds are the two solutions (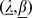 and 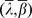) of the equations 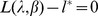 and 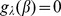, where 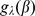 is given by (45), and 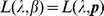 with 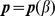 given by (44). A little algebraic manipulations yields that 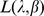 is given by (16e).

The solutions can be found by a Newton method. Straightforward calculation gives 
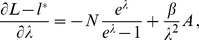


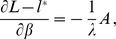


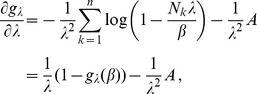


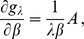
where 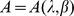 and 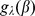 are given by (16c) and (45) or (16d), respectively. Hence, the Newton method leads to the following iteration



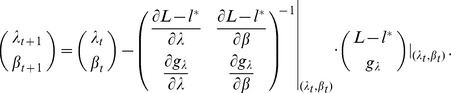



Due to its relatively simple form, the above matrix can be easily inverted and the iteration can be rewritten as (16a) and (16b).

The Newton methods converges locally quadratically if the above matrix is nonsingular in the solutions. Part A of the proof reveals that these solutions satisfy 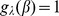, yielding 
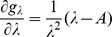
. Hence, the matrix simplifies to 
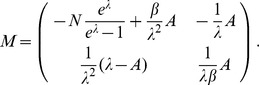



Therefore, 
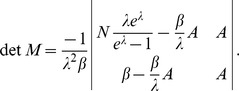



Clearly, since 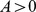, 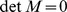 if and only if 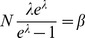. According to the proof of Result 1 this condition is only fulfilled at the unique ML estimate. Hence, 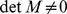 in 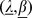 and 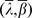. Therefore, the Newton method converges quadratically for any initial value sufficiently close to the respective solution.
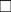


#### 2.3 Asymptotic confidence intervals. Proof of Result 3

This proof is slightly more general than necessary as we will re-use part of it later.

First, consider a matrix 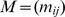 with the following structure 
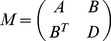
(50a)with



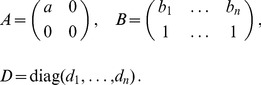
(50b)Let 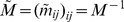. We aim to derive 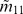. We do so by inverting 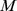 blockwise. Namely, 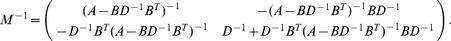



The formulae applies whenever, 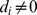 and the 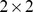 matrix 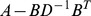 is invertible. Moreover, 
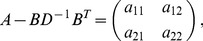
(51)where 
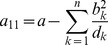
, 
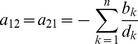
, and 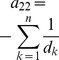. Its inverse is given by



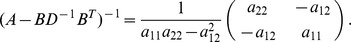



Hence, the desired quantity 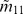 becomes 
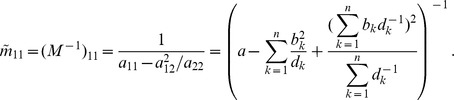



We are now ready to derive the confidence interval given by (18). To derive 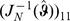 we first note that (7), (40) and rearrangement of the parameters imply that the Fisher information matrix has the form (50), with 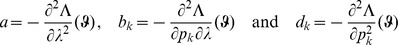
given by (40), and 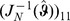 corresponds to 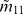. Therefore,
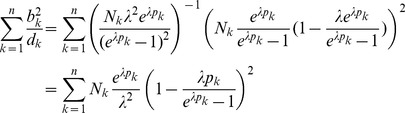
(52)and consequently



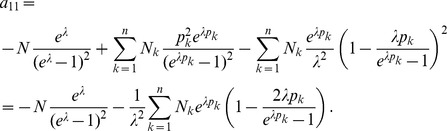



Moreover, 
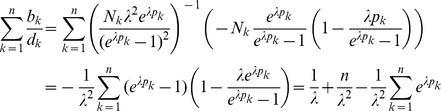
and



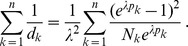



Hence, 
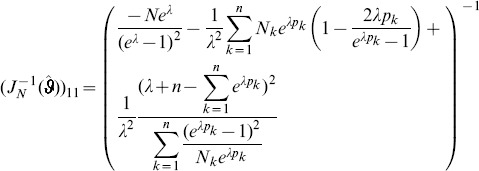
(53)


Deriving 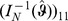 is easy. Namely, exactly the same calculations hold with 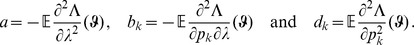



By inspecting (40), it becomes clear that all derivations remain unchanged with 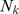 replaced by 
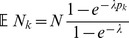
 (cf. eq. 53). This gives 
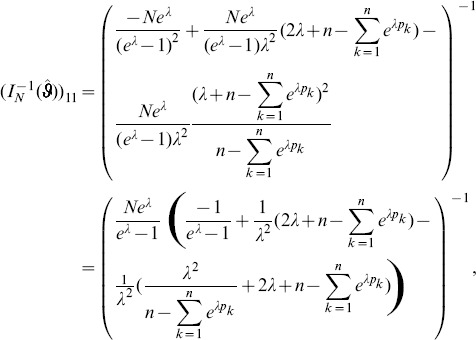
which simplifies to



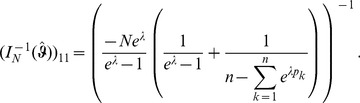
(54)Substituting the above with 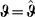 into (18) - using the fact that 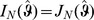 - yields (20) after after a little algebraic manipulation.

The identities 
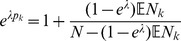
 follow from (43). Substituting this into (54) gives 
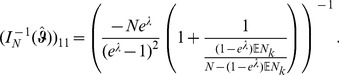
(55)


Substitution of the above evaluated at 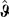 (using the fact that 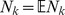) into (18) yields (21) after some rearrangement.

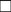



**Proof of Result 4.** To simplify the notation, we first derive the formulas for the confidence interval of 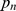. By re-arranging the parameters as in the proof of Result 3, it is obvious that the matrix 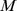 given by (50) can be used instead of the Fisher information 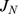 (or 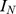). Particularly, 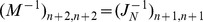.

We can apply a blockwise inversion formula to 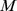 similar as in the proof of Result 3. Namely, 
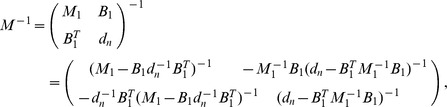
where



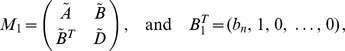
with



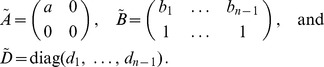



Clearly, 
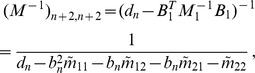
where 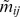 are the elements of 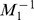. The inverse of 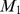 is calculated exactly as the inverse of 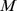 in the proof of Result 3. Namely, we arrive at



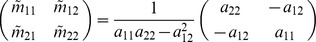
with 
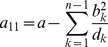
, 
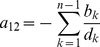
, and 
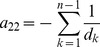
.

Hence, the desired quantity 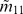 becomes 
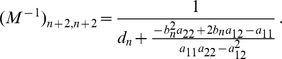



To derive the desired quantity 
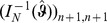
 (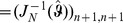) we need to set 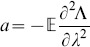, 
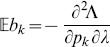
, and 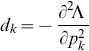. By using (40) and (43) we obtain 
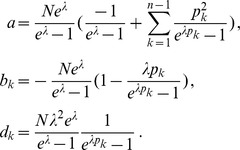



Therefore, 
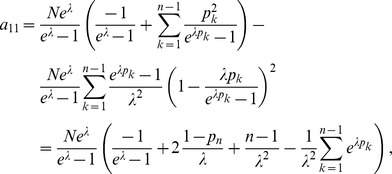


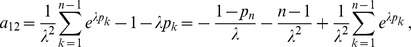


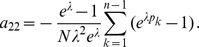



Hence, 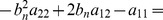


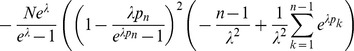


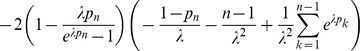


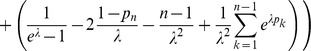


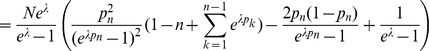



Moreover, 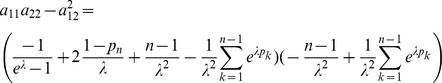


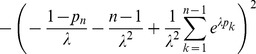


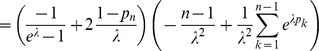


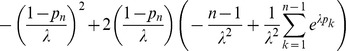


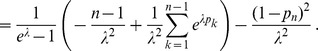



Combining the above yields, 
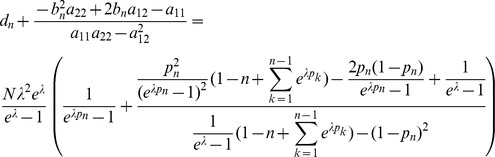
and finally



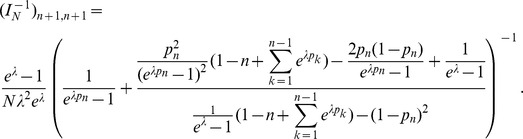



Hence, the bounds of the confidence intervals are given by 
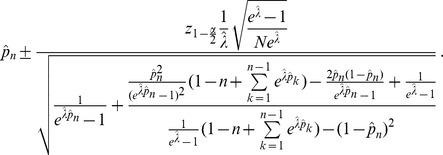



By replacing 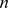 by 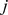, one obtains the confidence interval of 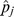 given by (22).
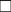



#### 2.4 Testing the Parameters

**Proof of Result 5.** The result is proven by showing that the iteration (29) leads to the profile-likelihood with 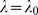. The proof of Remark 2 reveals that the desired values for 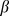 is the unique zero of 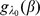 given by (45). The zero can be found using a Newton method. Combining (45) and (46) yields (29) after a little rearrangement.
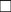



**Remark 6.** *If* 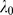 *and* 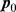 *(and* 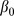 *) are the true (unknown) parameters, the asymptotic* 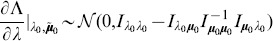 *holds.*

*We aim to test only for* 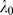 *, so any choice can be made for the true parameter. However, the parameters* 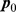 *occur in the asymptotic variance* 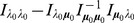 *. Hence, we need a plug-in estimate for the asymptotic variance. There are two possibilities. First, the true parameter* 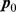 *is replaced by the profile-likelihood estimates* 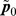 *based on* 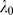 *and the asymptotic variance by* 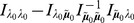 *. Here, either the expected or the observed Fisher information can be used.*

*Second, both* 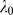 *and* 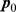 *can be replaced by the ML estimate* 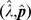 *. In this case the expected and observed Fisher information coincide.*

**Proof of Result 6.** The remark is proven by explicitly deriving the test statistic. To simplify the notation we write 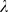 and 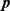 for 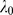 and 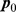, respectively. To derive 
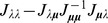
 (or 
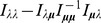
) we can follow the proof of Result 3.

From the blockwise inversion formula (51) the relation 
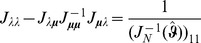
(56)follows immediately, where the denominator on the left-hand side is given by the reciprocal of (53).

Noting that 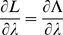 given by (39a) one obtains 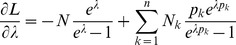. Substituting this and (56) in the test statistic (31), and writing 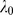 and 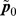 for 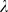 and 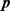 gives (32).

Of course, (56) also holds if 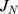 is replaced by 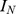, where 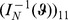 is given by (54). Thus, the same reasoning as above yields (33).
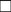


### **3 The case** 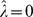


#### 3.1 Log-likelihood

In the limiting case that the true parameter is 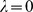 the conditional poison distribution becomes 
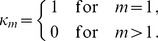
(57)


Following the derivations in subsection 4.1, 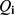 becomes 
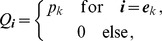
where 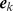 denotes the 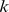
th base vector. Hence, the likelihood function (3) becomes



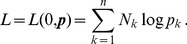
(58)This is the limiting case of (3) for 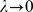. Furthermore, we can conclude the following.

**Remark 7.** *If the true parameter is* 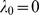 *, according to (57), an observation* 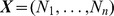 *with* 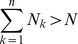 *is impossible in a sample of size* 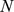 *. Hence,* 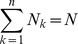 *with probability one.*

Assume 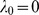 is the true parameter. Then, we can assume 
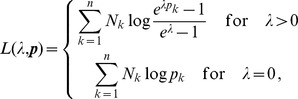



As mentioned above, the case 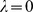 is just the continuation of the likelihood function.

Hence, we can define 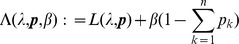. Moreover, the (one-sided) derivatives of the likelihood function 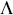 exist in 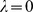. We have, 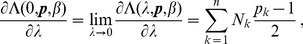
(59a)

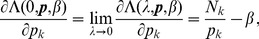
(59b)

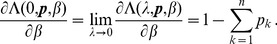
(59c)


The proof is found in the next subsection 6.2.

From (59a) we immediately see that 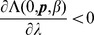. Hence, the ML estimate 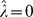 (cf. Remark 2) is a boundary maximum. However, it is necessary for the asymptotic distributions (11), (17), (30), and (38) that all derivatives of the likelihood function vanish. As this is not the case, we can neither derive confidence intervals, nor test the parameters in the case 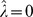.

### 3.2 Derivatives of the likelihood function



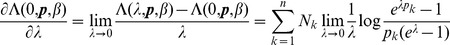



Applying de l'Hospitals rule gives 
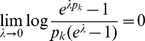
. Hence, successive application of this rule to the above yields 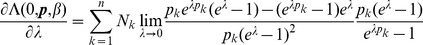


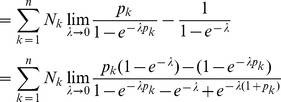


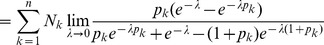


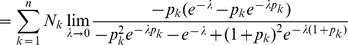


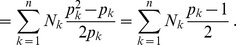



Note, that the last steps also proves 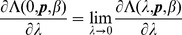.

Similarly, from (39d) 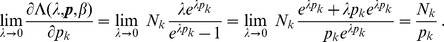



Since from (58) 
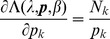
, one obtains (59b).
