# Correction: A Likelihood Approach to Estimate the Number of Co-Infections

**DOI:** 10.1371/journal.pone.0192877

**Published:** 2018-02-08

**Authors:** Kristan A. Schneider, Ananias A. Escalante

The authors wish to acknowledge an error that was overseen in the proof of Result 1 in [1]. By carefully inspecting the authors’ proof, it becomes clear that an MLE cannot exist if *N*_*k*_ = *N* for at least one *k*. This is easily seen from the authors’ formula for ${\hat{p}}_{k}$ in Result 1, where *N*_*k*_ = *N* implies ${\hat{p}}_{k}=1$, a contradiction since then $L(\lambda ,\hat{p}|x)=-\infty$. All results however remain valid as stated if their Assumption 1 is replaced by the following version.

**Assumption 1** *Assume that the sum over the lineages’ prevalences is larger than one*, *but no alleles is* 100% *prevalent. In other words, more than one lineage is found in at least one infection, i.e., $\sum_{k=1}^{n}N_{k}>N$ and all lineages are not found in every infection, i.e., N*_*k*_ ≠ *N for all k*.

By replacing Assumption 1 with the version above in [1], the results hold without modifications. All other modifications that need to be made in the article are minor and obvious. However, the case *N*_*k*_ = *N* for at least one *k* was not properly addressed. This occurred because it was overseen that the proof of in Result 1 is not applicable then. What goes wrong in this case? The answer is somewhat subtle. Heuristically, this contradiction occurs because no point in the parameter space is a critical point, i.e., a point at which all derivatives of *L* vanish. However, for any fixed *λ*, *L*(*λ*, ***p***|***x***) attains a maximum for some ${\hat{p}}^{\left(\lambda \right)}$, with $0<{\hat{p}}_{k}^{\left(\lambda \right)}<1$. The reason is that *L*(*λ*, ***p***|***x***) = −∞ for $p\in bdS_{n}$ (where $S_{n}$ denotes the *n*−1-dimensional simplex). For *λ* → 0, $L(\lambda ,{\hat{p}}^{\left(\lambda \right)}|x)\to -\infty$. Hence, $L(\lambda ,{\hat{p}}^{\left(\lambda \right)}|x)$ is necessarily monotonically increasing in *λ*, implying that no MLE exists. In mathematical terms this can be formulated as follows:

**Remark 1** *Assume that at least one lineage is found in every sample*, *i*.*e*.,*N*_*k*_ = *N for at least one k*, *but not all are found in every sample*, *i*.*e*.,*N*_*k*_ ≠ *N for at least one j*. *Then*, *the log-likelihood function does not attain a maximum*. *However*, *its smallest upper bound is*

$$\underset{p\in S_{n},\lambda >0}{\text{sup}}L(\lambda ,p|x)=\sum_{\begin{array}{c} k=1\\ N_{k}\neq N \end{array}}^{n}(N-N_{k})\text{log}(1-\frac{N_{k}}{N}).$$

*The supremum is reached in the limit of any sequence* (*λ*_*t*_, ***p***_*t*_) *with* $\underset{t\to \infty }{\text{lim}}\lambda_{t}=\infty$, $\underset{t\to \infty }{\text{lim}}p_{k}^{\left(t\right)}=-\text{log}\left(1-\frac{N_{k}}{N}\right)$ *if N*_*k*_ ≠ *N and* $\underset{t\to \infty }{\text{lim}}p_{k}^{\left(t\right)}\lambda_{t}=\infty$ if *N*_*k*_ = *N*.

**Proof**. Because *L*(*λ*, ***p***|***x***) is bounded by 0, the supremum exists. Furthermore, a sequence (*λ*_*t*_, ***p***_*t*_) exists with $L(\lambda_{t},p_{t}|x)\to \underset{p\in S_{n},\lambda >0}{\text{sup}}L(\lambda ,p|x)$.

Without loss of generality let *N*_1_,…,*N*_*m*_ < *N* and *N*_*m*+1_ = … = *N*_*n*_ = *N*. Hence,

$$\begin{array}{ll} L(\lambda ,p|x) & =-N\text{log}(e^{\lambda }-1)+\sum_{k=1}^{n}N_{k}\text{log}(e^{\lambda p_{k}}-1)\\ & =N\text{log}\frac{\left(e^{\lambda p_{m+1}}-1)\cdot \ldots \cdot (e^{\lambda p_{n}}-1\right)}{e^{\lambda }-1}+\sum_{k=1}^{m}N_{k}\text{log}(e^{\lambda p_{k}}-1)\\ & =N\text{log}\frac{\left(1-e^{-\lambda p_{m+1}})\cdot \ldots \cdot (1-e^{-\lambda p_{n}}\right)}{1-e^{-\lambda }}e^{-\lambda (1-p_{m+1}-\ldots -p_{n})}+\sum_{k=1}^{m}N_{k}\text{log}(e^{\lambda p_{k}}-1)\\ & =N\text{log}\frac{\left(1-e^{-\lambda p_{m+1}})\cdot \ldots \cdot (1-e^{-\lambda p_{n}}\right)}{1-e^{-\lambda }}e^{-\lambda (p_{1}+\ldots +p_{m})}+\sum_{k=1}^{m}N_{k}\text{log}(e^{\lambda p_{k}}-1). \end{array}$$ (1)

Let (*λ*_*t*_) be any monotone sequence with $\underset{t\to \infty }{\text{lim}}\lambda_{t}=\infty$. Moreover, let *c*_*k*_ >0 for *k* = 1,…,*m*. Now let ***p***_*t*_ be a sequence satisfying $\underset{t\to \infty }{\text{lim}}p_{k}^{\left(t\right)}\lambda_{t}=c_{k}$ for *k* = 1,…,*m* and $\underset{t\to \infty }{\text{lim}}p_{k}^{\left(t\right)}\lambda_{t}=\infty$ for *k* = *m* + 1,…,*n*. Without loss of generality let $p_{k}^{\left(t\right)}=\frac{c_{k}}{\lambda_{t}}$ for *k* = 1,…,*m* and $p_{k}^{\left(t\right)}=\frac{1}{n-m}(1-\sum_{k=1}^{m}\frac{c_{k}}{\lambda_{t}})$ for *k* = *m* + 1,…,*n*. For sufficiently large *t* this sequence is defined and $p_{t}\in S_{n}$. Hence,

$$\begin{array}{c} \underset{t\to \infty }{\text{lim}}\;L(\lambda_{t},p_{t}|x)=N\text{log}1\cdot e^{-c_{1}-\ldots -c_{m}}+\sum_{k=1}^{m}N_{k}\text{log}(e^{c_{k}}-1)\\ \;=-N(c_{1}+\ldots +c_{m})+\sum_{k=1}^{m}N_{k}\text{log}(e^{c_{k}}-1). \end{array}$$

Next define $f(c_{1},\ldots ,c_{m}):=\underset{t\to \infty }{\text{lim}}\;L(\lambda_{t},p_{t}|x)$. Note that this definition is independent of the sequence (*λ*_*t*_, ***p***_*t*_), with *λ*_*t*_***p***_*t*_ →(*c*_1_,…,*c*_*m*_, ∞,…,∞) for *t* → ∞.

The next aim, is to identify potential maxima of *f*. Clearly, $\frac{\partial f}{\partial c_{k}}=-N+N_{k}\frac{e^{c_{k}}}{e^{c_{k}}-1}$. Equating the partial derivatives to zero gives ${\hat{c}}_{k}=-\text{log}(1-\frac{N_{k}}{N})$. The Hessian matrix is given by $H=-\text{diag}{\left(N_{k}\frac{e^{c_{k}}}{{\left(e^{c_{k}}-1\right)}^{2}}\right)}_{k=1,\ldots ,n}$ and clearly negative definite. Thus, *f* attains a global maximum at ${\hat{c}}_{k}=-\text{log}(1-\frac{N_{k}}{N})$. Therefore $f({\hat{c}}_{1},\ldots ,{\hat{c}}_{m})\leq \underset{p\in S_{n},\lambda >0}{\text{sup}}L(\lambda ,p|x)$.

If (*λ*_*t*_, ***p***_*t*_) is any sequence with $\lambda_{t}p_{k}^{\left(t\right)}\to \infty$ for a *k* with 1 ≤ *k* ≤ *m*, it is easily seen from (1) that $\underset{t\to \infty }{\text{lim}}\;L(\lambda_{t},p_{t}|x)=-\infty$. Moreover, if $\lambda_{t}p_{k}^{\left(t\right)}\to c_{k}<\infty$ for 1 ≤ *k* ≤ *m* and at least one *k* with *m* + 1 ≤ *k* ≤ *n*, without loss of generality $\lambda_{t}p_{k}^{\left(t\right)}\to c_{k}<\infty$ for m+1≤k≤l, (1) implies

$$\underset{t\to \infty }{\text{lim}}L(\lambda_{t},p_{t}|x)=-N(c_{1}+\cdots +c_{m})+\sum_{k=1}^{m}N_{k}\text{log}(e^{c_{k}}-1)+N\sum_{k=m+1}^{l}\text{log}\left(1-e^{{-c}_{k}}\right)<-N(c_{1}+\cdots +c_{m})+\sum_{k=1}^{m}N_{k}\text{log}\left(e^{c_{k}}-1\right)$$

implying that this limit is less than the maximum of *f*. The above considerations imply that the supremum of the log-likelihood function must be the maximum of *f*. Deriving $f({\hat{c}}_{1},\ldots ,{\hat{c}}_{m})$ finishes the proof.

The case that *N*_*k*_ = *N* for all *k* is treated in [1]. Moreover, obviously in Remark 1 of [1] a misprint occurred. The expression $\sum_{k=1}^{n}N_{k}\geq N$ needs to be replaced by $\sum_{k=1}^{n}N_{k}>N$, while the same expression needs to be replaced by $\sum_{k=1}^{n}N_{k}=N$ in the paragraph below Result 2.
